# Supplementary material for: Design, Synthesis, Kinetic Analysis and Pharmacophore-Directed Discovery of 3-Ethylaniline Hybrid Imino-Thiazolidinone as Potential Inhibitor of Carbonic Anhydrase II: An Emerging Biological Target for Treatment of Cancer
Source: Biomolecules. 2022 Nov 16;12(11):1696. doi: 10.3390/biom12111696 (PMC9687900; doi:10.3390/biom12111696)
Supplement: Supplementary file 1 [file biomolecules-12-01696-s001.zip › biomolecules-2002973-supplementary.pdf]

Supporting information

Atteeque Ahmed[1], Mubashir Aziz[2] Syeda Abida Ejaz[2*], Pervaiz Ali Channar[3], Aamer Saeed[1*], Seema Zargar[4], Tanveer A. Wani[5], Asad Hamad,[6] Qamar Abbas[7], Hussain Raza[8], Song Ja Kim[8]

[1]*Department of Chemistry, Quaid-I-Azam University, Islamabad 45320, Pakistan.*

[2]*Department of Pharmaceutical Chemistry, Faculty of Pharmacy, The Islamia University of Bahawalpur, Bahawalpur-63100, Pakistan.*

[3]*Department of Basic Sciences and Humanities, Faculty of of Information Science and Humanities.Dawood University of Engineering and Technology, Karachi 74800, Pakistan*

[4]*Department of Biochemistry, College of Science, King Saud University, P.O. Box 22452,*

Riyadh 11451, Saudi Arabia

[5]*Department of Pharmaceutical Chemistry, College of Pharmacy, King Saud University, P.O.*

Box 2457, Riyadh 11451, Saudi Arabia

[6] *Faculty of Pharmacy, Grand Asian University Sialkot, 51310, Punjab, Pakistan.*

[7]*Department of Biology, College of Science, University of Bahrain, Sakhir-32038, Kingdom of Bahrain.*

[8]*College of Natural Sciences, Department of Biological Sciences, Kongju National University, Gongju-32588, Republic of Korea.*

Corresponding Authors:

Aamer Saeed; Email: asaeed@qau.edu.pk, aamersaeed@yahoo.com;

Syeda Abida Ejaz; abida.ejaz@iub.edu.pk; abidaejaz2010@gmail.com

To whom correspondences should address:

Dr. Syeda Abida Ejaz; Department of Pharmaceutical Chemistry, Faculty of Pharmacy, The Islamia University of Bahawalpur, Pakistan., Postal Code 63100, Pakistan Tel: +92-062-9250245 Fax: +92-062-9250245, E-Mail: abida.ejaz@iub.edu.pk; abidaejaz2010@gmail.com

Ethyl(Z)-2-((Z)-3-(3-ethylphenyl)-2-((2-methylbenzoyl)imino)-4-oxothiazolidin-5-ylidene)acetate (6a)

White solid Yield: 83%; $R_{f;}$ 0.61: m.p. 235-237°C; **FTIR:** 2974.45 (C-H aliphatic, asymmetric, sp^3), 2928.12 (C-H aliphatic, symmetric, sp^3), 1733.34, 1695.13, 1649.57 (3C=O), 1538.82 (N=C), 1191 (C-S) cm^{-1}. **^{1}H NMR** (300 MHz, $CDCl_3$) δ 7.96 (dd, *J* = 7.8, 1.5 Hz, 1H), 7.49 (t, *J* = 7.7 Hz, 1H), 7.38 (qd, *J* = 6.8, 3.0 Hz, 2H), 7.23 (dq, *J* = 8.0, 3.2 Hz, 3H), 7.16 (t, *J* = 7.9 Hz, 1H), 7.09 (s, 1H), 4.38 (q, *J* = 7.1 Hz, 2H, CH_2), 2.77 (q, *J* = 7.6 Hz, 2H, CH_2), 2.61 (s, 3H, CH_3), 1.36 (dt, *J* = 25.0, 7.4 Hz, 6H, $(CH_3)_2$). **^{13}C NMR** (75 MHz, $CDCl_3$) δ 178.3 (C=O), 165.2(C=O), 165.0(C=O), 164.8, 145.6, 141.6, 140.9, 134.1, 133.4, 132.7, 132.1, 131.9, 129.1, 129.0, 127.1, 125.6, 124.8, 120.6, 61.9(CH_2), 28.6(CH_2), 22.4(CH_3), 15.3(CH_3), 14.2(CH_3). Anal. calcd. For $C_{23}H_{22}N_2O_4S$ (422.13): C, 65.39, H, 5.25, N, 6.63, O, 15.15, S, 7.59. Found: C, 65.37, H, 5.22, N, 6.60, S, 7.57. GC-MS (ESI^-) m/z = 422.0.

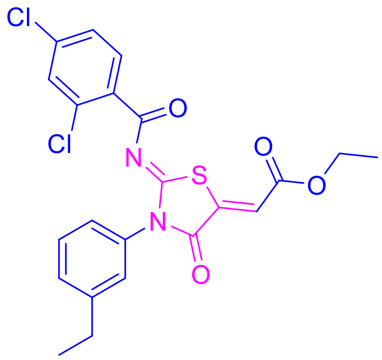

Ethyl(Z)-2-((Z)-2-((2,4-dichlorobenzoyl)imino)-3-(3-ethylphenyl)-4-oxothiazolidin-5-ylidene)acetate (6b)

White solid Yield: 87%; $R_{f;}$ 0.49: m.p. 235-237°C; **FTIR:** 2971.62 (C-H aliphatic, asymmetric, sp^3), 2929.47 (C-H aliphatic, symmetric, sp^3), 1736.53, 1702.44, 1649.79 (3C=O), 1532.63 (N=C), 1185.35 (C-S) cm^{-1}. **^{1}H NMR** (300 MHz, $CDCl_3$) δ 7.85 (d, *J* = 8.5 Hz, 1H), 7.45 (d, *J* = 8.5 Hz,

2H), 7.39 – 7.25 (m, 1H), 7.24 – 7.08 (m, 2H), 4.38 (q, *J* = 7.2 Hz, 2H, CH_2), 2.75 (q, *J* = 7.7 Hz, 2H, CH_2), 1.35 (dt, *J* = 29.2, 7.4 Hz, 6H, $(CH_3)_2$). **^{13}C NMR** (75 MHz, $CDCl_3$) δ 175.1(C=O), 167.2(C=O), 165.0(C=O), 164.9, 145.6, 140.2, 138.7, 135.8, 133.9, 133.8, 131.7, 131.2, 129.1, 127.0, 126.8, 124.8, 121.5, 62.1(CH_2), 28.6(CH_2), 15.3(CH_3), 14.2(CH_3). Anal. calcd. For $C_{22}H_{18}Cl_2N_3O_4S$ (476.03): C, 55.36, H, 3.80, Cl, 14.85, N, 5.87, O, 13.41, S, 6.72. Found: C, 55.33, H, 3.79, N, 5.85, S, 6.72. GC-MS (ESI^-) m/z = 476.01

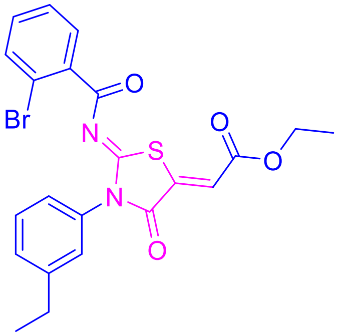

Ethyl(Z)-2-((Z)-2-((3-bromobenzoyl)imino)-3-(3-ethylphenyl)-4-oxothiazolidin-5-ylidene)acetate (6c)

White solid Yield: 80%; $R_{f;}$ 0.55: m.p. 235-237°C; **FTIR:** 2966.12 (C-H aliphatic stretch), 1686.18(C=O), 1538.82 (N=C), 1156.93 (C-S) cm^{-1} .

^{1}H NMR (300 MHz, DMSO-d_6) δ 7.64 – 7.55 (m, 1H), 7.44 (t, *J* = 8.1 Hz, 1H), 7.33 (dd, *J* = 5.8, 3.5 Hz, 3H), 7.14 (d, *J* = 6.0 Hz, 2H), 7.00 – 6.88 (m, 1H), 6.78 (s, 1H), 4.25 (q, *J* = 7.0 Hz, 2H, CH_2), 2.64 (q, *J* = 7.6 Hz, 2H, CH_2), 1.23 (dt, *J* = 31.4, 7.4 Hz, 6H, $(CH_3)_2$). **^{13}C NMR** (75 MHz, DMSO-d_6) δ 166.5(C=O), 166.4(C=O), 163.4(C=O), 147.2, 145.1, 137.4, 134.8, 133.3, 131.9, 129.5, 129.4, 128.6, 127.7, 127.4, 125.5, 119.3, 112.9, 106.5, 61.5(CH_2), 51.3(CH_2), 15.9(CH_3), 14.5(CH_3). Anal. calcd. For $C_{22}H_{19}BrN_2O_4S$ (486.0249): C, 54.22, H, 3.93, Br, 16.40, N, 5.75, O, 13.13, S, 6.58. Found: C, 54.20, H, 3.90, N, 5.73, S, 6.53. GC-MS (ESI^-) m/z = 486.0

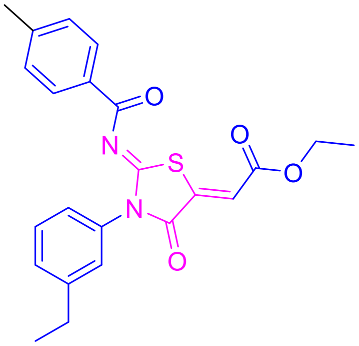

Ethyl(Z)-2-((Z)-3-(3-ethylphenyl)-2-((4-methylbenzoyl)imino)-4-oxothiazolidin-5-ylidene)acetate(6d)

White solid Yield: 85%; $R_{f;}$ 0.57: m.p. 235-237°C; **FTIR:** 2955.61 (C-H aliphatic stretch), 1736.18, 1702.71, 1640.34(3C=O), 1537.10 (N=C), 1163.83 (C-S) cm^{-1} .**^{1}H NMR** (300 MHz, $CDCl_3$) δ 7.99 – 7.89 (m, 2H), 7.51 (t, *J* = 7.7 Hz, 1H), 7.37 (dt, *J* = 7.8, 1.3 Hz, 1H), 7.30 – 7.15 (m, 4H), 7.08 (s, 1H), 4.37 (q, *J* = 7.1 Hz, 2H), 2.78 (q, *J* = 7.6 Hz, 2H, CH_2), 2.40 (s, 3H, CH_2), 1.36 (dt, *J* = 20.6, 7.4 Hz, 6H, $(CH_3)_2$). **^{13}C NMR** (75 MHz, $CDCl_3$) δ 176.8(C=O), 165.7(C=O), 165.1(C=O), 165.1, 145.5, 144.5, 140.8, 134.0, 132.1, 130.4, 129.1, 129.1, 129.0, 127.1, 124.8, 120.8, 120.3, 77.2, 62.0, 52.8(CH_2), 28.7(CH_2), 21.8(CH_3), 15.4(CH_3), 14.2(CH_3). Anal. calcd. For $C_{23}H_{22}N_2O_4S$ (422.13): C, 65.39, H, 5.25, N, 6.63, O, 15.15, S, 7.59. Found: C, 65.37, H, 5.22, N, 6.60, S, 7.57. GC-MS (ESI^-) m/z = 422.0.

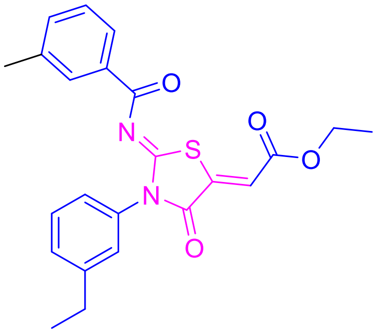

Ethyl(Z)-2-((Z)-3-(3-ethylphenyl)-2-((3-methylbenzoyl)imino)-4-oxothiazolidin-5-ylidene)acetate (6e)

White solid Yield: 79%; $R_{f;}$ 0.59: m.p. 235-237°C; **FTIR:** 2961.35 (C-H aliphatic, asymmetric, sp^3), 2925.447 (C-H aliphatic, symmetric, sp^3), 1734.11, 1695.79, 1644.37 (3C=O), 1523.49 (N=C), 1159.61 (C-S) cm^{-1} .**^{1}H NMR** (300 MHz, $CDCl_3$) δ 7.92 – 7.79 (m, 2H), 7.51 (t, *J* = 7.7 Hz, 1H), 7.43 – 7.19 (m, 5H), 7.09 (s, 1H), 4.38 (q, *J* = 7.1 Hz, 2H, CH_2), 2.78 (q, *J* = 7.6 Hz, 2H, CH_2), 2.36 (s, 3H, CH_3), 1.37 (dt, *J* = 20.2, 7.4 Hz, 6H, $(CH_3)_2$). **^{13}C NMR** (75 MHz, $CDCl_3$) δ 177.1(C=O), 165.9(C=O), 165.1(C=O), 165.1, 145.5, 140.7, 138.1, 134.6, 134.4, 133.9, 130.8, 129.1, 129.0, 128.3, 127.6, 127.1, 124.8, 120.9, 62.0(CH_2), 28.7(CH_2), 21.3(CH_3), 15.4(CH_3),

14.2(CH₃). Anal. calcd. For $C_{23}H_{22}N_2O_4S$ (422.13): C, 65.39, H, 5.25, N, 6.63, O, 15.15, S, 7.59. Found: C, 65.37, H, 5.22, N, 6.60, S, 7.57. GC-MS (ESI$^-$) m/z = 422.0.

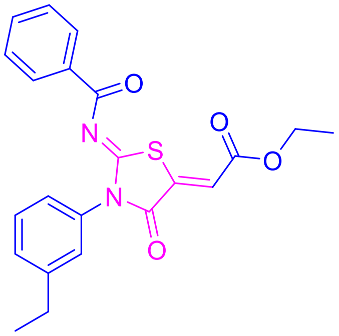

Ethyl(Z)-2-((Z)-2-((3-benzoyl)imino)-3-(3-ethylphenyl)-4-oxothiazolidin-5-ylidene)acetate (6f)

White solid Yield: 81%; $R_{f;}$ 0.61: m.p. 235-237°C; **FTIR:** 2960.56 (C-H aliphatic stretch), 1737.20, 1697.48, 1637.27 (3C=O), 1513.26 (N=C), 1186.30 (C-S) cm^{-1} .^{1}**H NMR** (300 MHz, $CDCl_3$) δ 7.49 (td, *J* = 9.3, 4.7 Hz, 2H), 7.37 (s, 1H), 7.31 – 7.05 (m, 5H), 4.37 (q, *J* = 7.1 Hz, 2H), 2.76 (q, *J* = 7.6 Hz, 2H), 1.35 (dt, *J* = 24.6, 7.4 Hz, 6H). Anal. calcd. For $C_{22}H_{20}N_2O_4S$ (408.1144): C, 64.69, H, 4.94, N, 6.86, O, 15.67, S, 7.85. Found: C, 64.67, H, 4.92, N, 6.83, S, 7.84.GC-MS (ESI$^-$) m/z = 408.0.

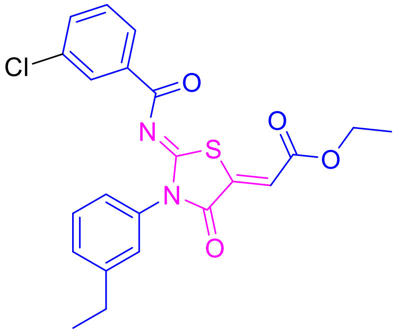

Ethyl(Z)-2-((Z)-2-((3-chlorobenzoyl)imino)-3-(3-ethylphenyl)-4-oxothiazolidin-5-ylidene)acetate (6g)

White solid Yield: 86%; $R_{f;}$ 0.52: m.p. 235-237°C; **FTIR:** 2960.56 (C-H aliphatic stretch), 1737.20, 1697.48, 1637.27 (3C=O), 1513.26 (N=C), 1186.30 (C-S) cm^{-1} .^{1}**H NMR** (300 MHz, $CDCl_3$) δ 8.04 – 7.87 (m, 1H), 7.51 (t, *J* = 7.8 Hz, 1H), 7.43 – 7.18 (m, 2H), 4.38 (q, *J* = 7.1 Hz, 1H, CH_2), 2.79 (q, *J* = 7.6 Hz, 1H, CH_2), 1.37 (dt, *J* = 18.5, 7.4 Hz, 3H, $(CH_3)_2$). ^{13}**C NMR** (75 MHz, $CDCl_3$) δ 175.7(C=O), 167.4(C=O), 165.0(C=O), 164.9, 145.7, 140.3, 136.5, 134.5, 133.8,

133.4, 130.3, 129.7, 129.1, 129.1, 128.3, 127.0, 124.7, 121.4, 62.1(CH_2), 28.7(CH_2), 15.4(CH_3), 14.2(CH_3). Anal. calcd. For $C_{22}H_{19}ClN_2O_4S$ (442.0754): C, 59.66, H, 4.32, Cl, 8.00, N, 6.32, O, 14.45, S, 7.24. Found: C, 59.62, H, 4.30, N, 6.29, S, 7.21. GC-MS (ESI^-) m/z = 442.0.

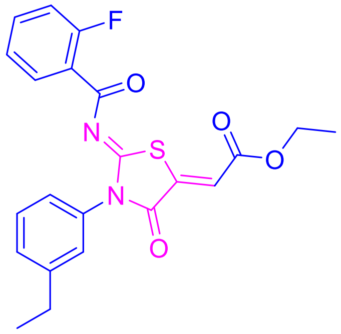

Ethyl(Z)-2-((Z)-3-(3-ethylphenyl)-2-((2-fluorobenzoyl)imino)-4-oxothiazolidin-5-ylidene)acetate (6h)

White solid Yield: 78%; $R_{f;}$ 0.50: m.p. 235-237°C;**FTIR:** 2970.41 (C-H aliphatic stretch), 1731.79, 1692.17, 1651.13 (3C=O), 1530.36 (N=C), 1192.24 (C-S) cm^{-1} .**^{1}H NMR** (300 MHz, $CDCl_3$) δ 7.91 (td, J = 8.0, 2.0 Hz, 1H), 7.49 (td, J = 9.3, 4.7 Hz, 2H), 7.37 (s, 1H), 7.31 – 7.05 (m, 5H), 4.37 (q, J = 7.1 Hz, 2H, CH_2), 2.76 (q, J = 7.6 Hz, 2H, CH_2), 1.35 (dt, J = 24.6, 7.4 Hz, 5H, $(CH_3)_2$). **^{13}C NMR** (75 MHz, $CDCl_3$) δ 174.6(C=O), 174.6(C=O), 165.9(C=O), 165.0, 164.9, 164.5, 161.0, 145.6, 140.4, 135.1, 135.0, 133.8, 132.9, 129.1, 129.0, 127.1, 124.8, 123.8, 123.8, 123.0, 122.9, 121.2, 117.2, 116.9, 77.2, 62.0(CH_2), 28.6(CH_2), 15.3(CH_3), 14.2(CH_3). Anal. calcd. For $C_{22}H_{19}FN_2O_4S$ (426.1050): C, 61.96, H, 4.49, F, 4.45, N, 6.57, O, 15.01, S, 7.52. Found: C, 61.94, H, 4.46, N, 6.55, S, 7.50. GC-MS (ESI^-) m/z = 426.0.

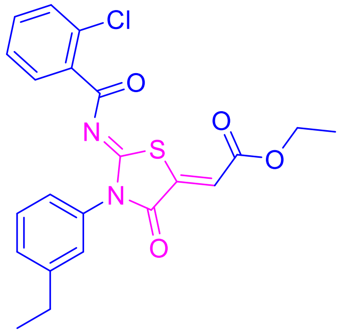

Ethyl(Z)-2-((Z)-2-((2-chlorobenzoyl)imino)-3-(3-ethylphenyl)-4-oxothiazolidin-5-ylidene)acetate (6i)

White solid Yield: 80%; R_f; 0.54: m.p. 235-237°C; **FTIR:** 2971.41 (C-H aliphatic stretch), 1731.31, 1693, 1650.20 (3C=O), 1524.38 (N=C), 1187.81 (C-S) cm^{-1} .**^{1}H NMR** (300 MHz, $CDCl_3$) δ 7.94 – 7.85 (m, 1H), 7.53 – 7.15 (m, 7H), 4.38 (q, *J* = 7.1 Hz, 2H, CH_2), 2.75 (q, *J* = 7.6 Hz, 2H, CH_2), 1.39 (t, *J* = 7.1 Hz, 3H, CH_3), 1.30 (t, *J* = 7.6 Hz, 3H, CH_3). **^{13}C NMR** (75 MHz, $CDCl_3$) δ 176.2(C=O), 166.5(C=O), 165.0(C=O), 165.0, 145.6, 140.3, 134.7, 133.8, 133.3, 133.1, 132.9, 131.4, 129.1, 129.1, 127.1, 126.4, 124.8, 121.3, 62.1(CH_2), 28.6(CH_2), 15.3(CH_3), 14.2(CH_3). Anal. calcd. For $C_{22}H_{19}ClN_2O_4S$ (442.0754): C, 59.66, H, 4.32, Cl, 8.00, N, 6.32, O, 14.45, S, 7.24. Found: C, 59.62, H, 4.30, N, 6.29, S, 7.21. GC-MS (ESI^-) m/z = 442.0.

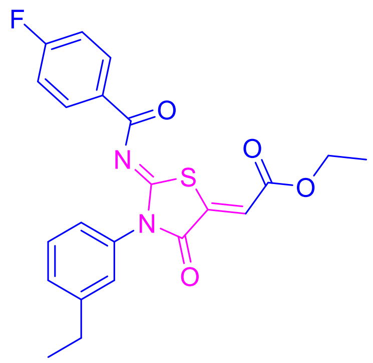

Ethyl(Z)-2-((Z)-3-(3-ethylphenyl)-2-((4-fluorobenzoyl)imino)-4-oxothiazolidin-5-ylidene)acetate (6j)

White solid Yield: 85%; R_f; 0.47: m.p. 235-237°C; **FTIR:** 2966.32 (C-H aliphatic stretch), 1731.46, 1699.03, 1644.40 (3C=O),1527.99 (N=C), 1186.45 (C-S) cm^-1.**1H NMR** (300 MHz, $CDCl_3$) δ 8.12 – 8.00 (m, 2H), 7.51 (t, *J* = 7.7 Hz, 1H), 7.33 – 7.17 (m, 2H), 7.13 – 6.99 (m, 3H), 4.38 (q, *J* = 7.1 Hz, 2H, CH_2), 2.78 (q, *J* = 7.6 Hz, 2H, CH_2), 1.36 (dt, *J* = 22.8, 7.4 Hz, 6H, $(CH_3)_2$). **^{13}C NMR** (75 MHz, $CDCl_3$) δ 175.7(C=O), 167.8(C=O), 166.7(C=O), 165.1, 165.0, 164.4, 145.6, 140.5, 133.9, 133.0, 132.9, 131.1, 131.1, 129.1, 129.1, 127.0, 124.8, 121.2, 115.6, 115.3, 62.0(CH_2), 28.7(CH_2), 15.4(CH_3), 14.2(CH_3). Anal. calcd. For $C_{22}H_{19}FN_2O_4S$ (426.1050): C, 61.96, H, 4.49, F, 4.45, N, 6.57, O, 15.01, S, 7.52. Found: C, 61.94, H, 4.46, N, 6.55, S, 7.50. GC-MS (ESI^-) m/z = 426.0.

s

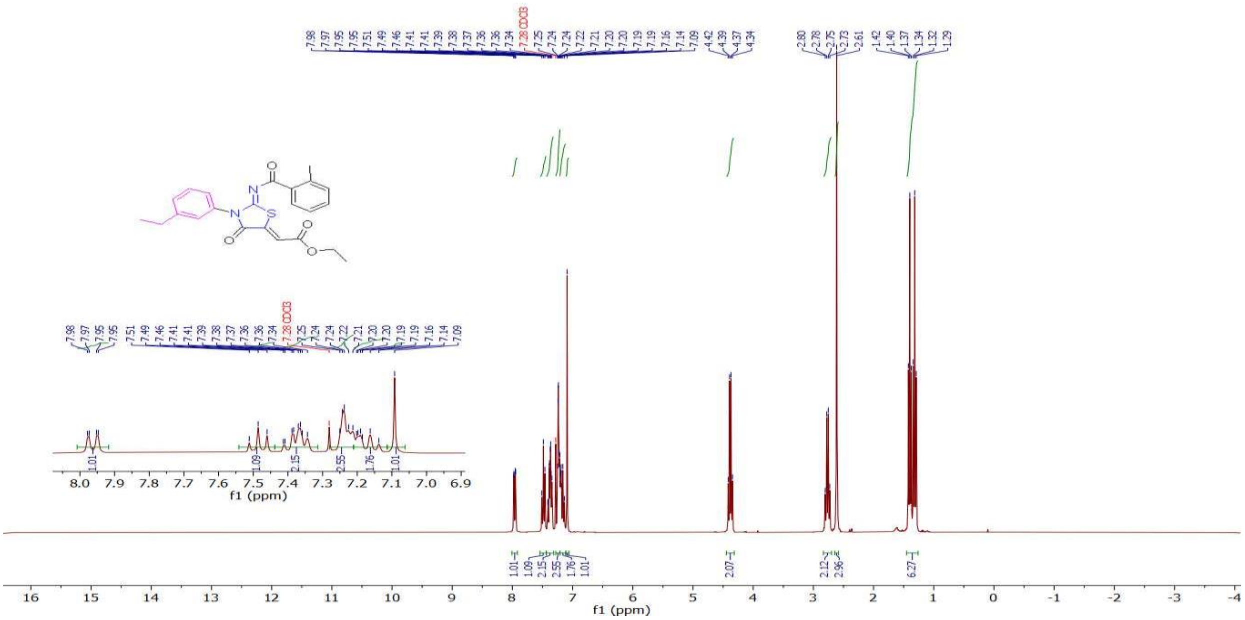

Figure S1 ***^{1}HNMR of Ethyl(Z)-2-((Z)-3-(3-ethylphenyl)-2-((2-methylbenzoyl)imino)-4-oxothiazolidin-5-ylidene)acetate (6a)***

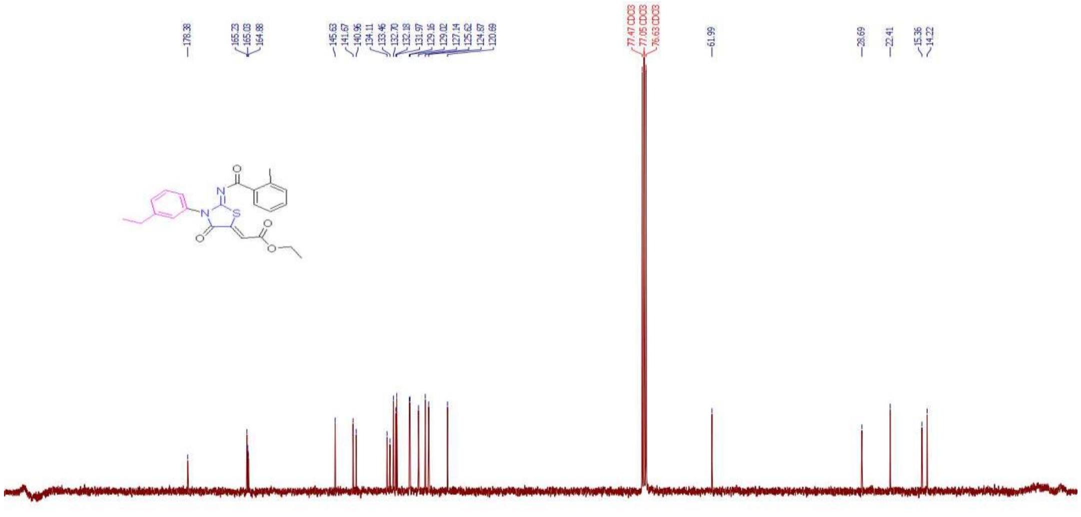

Figure S2 ***^{13}CNMR Ethyl(Z)-2-((Z)-3-(3-ethylphenyl)-2-((2-methylbenzoyl)imino)-4-oxothiazolidin-5-ylidene)acetate (6a)***

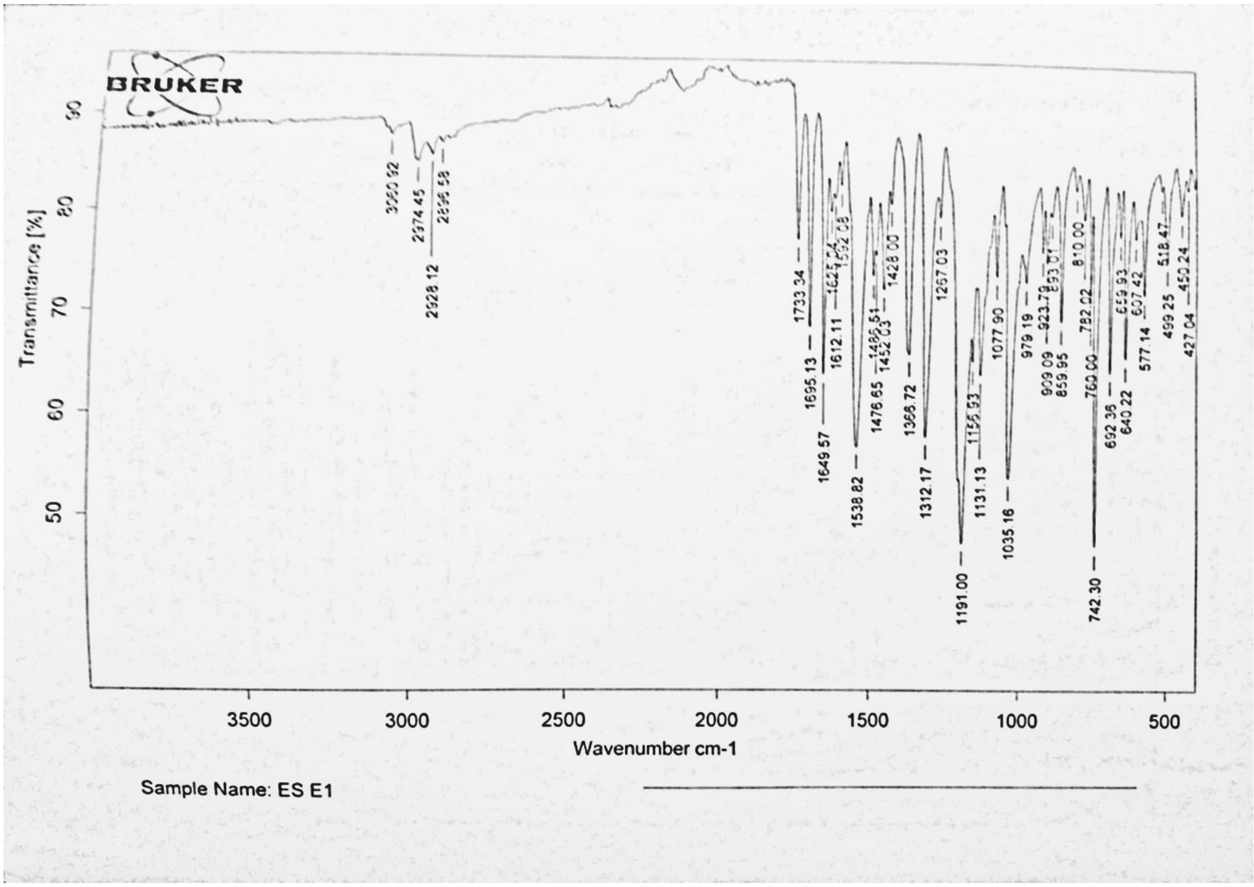

Figure S3 IR spectra of Ethyl(Z)-2-((Z)-3-(3-ethylphenyl)-2-((2-methylbenzoyl)imino)-4-oxothiazolidin-5-ylidene)acetate (6a)

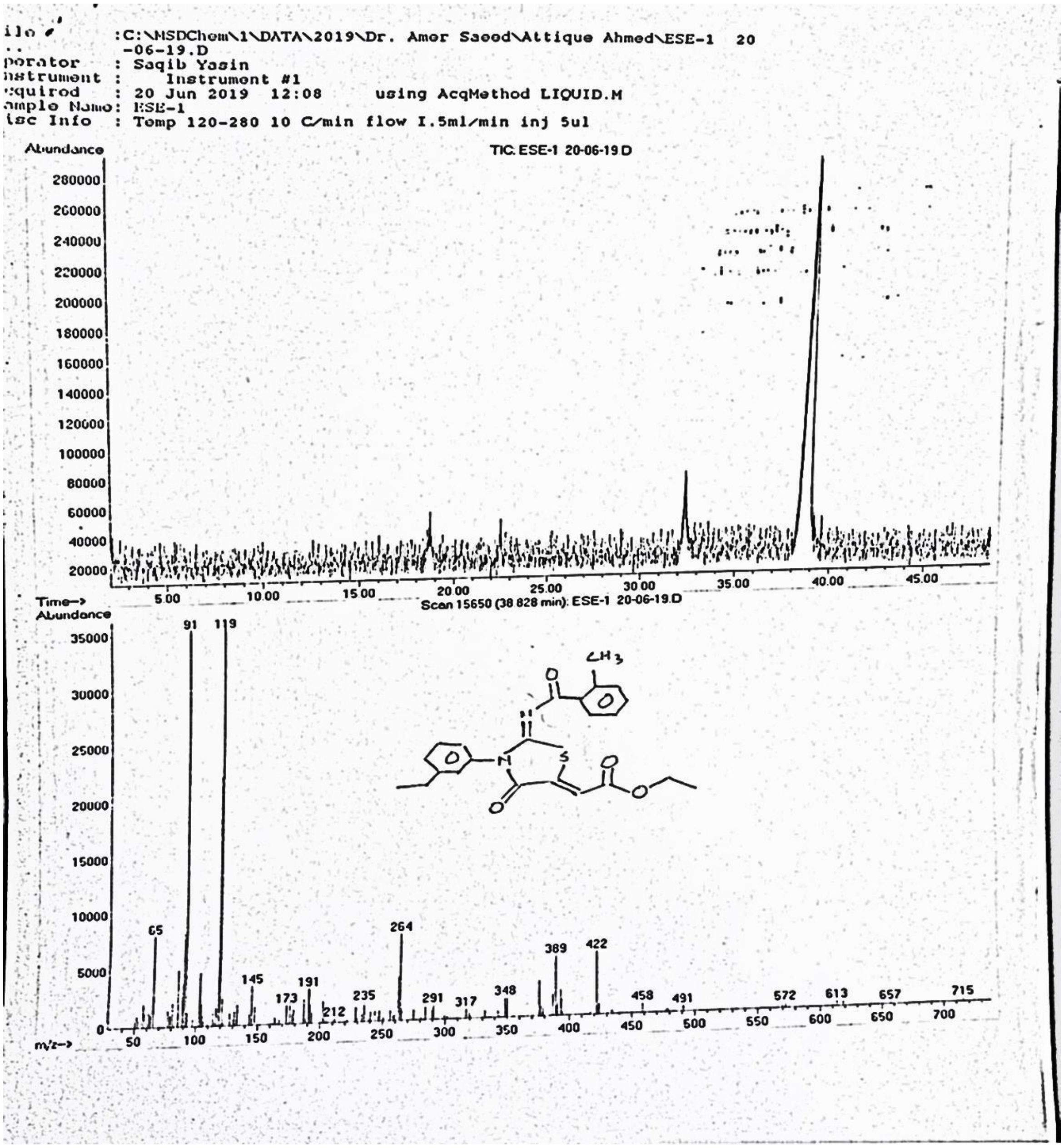

Figure S4 MS spectra of Ethyl(Z)-2-((Z)-3-(3-ethylphenyl)-2-((2-methylbenzoyl)imino)-4-oxothiazolidin-5-ylidene)acetate (6a)

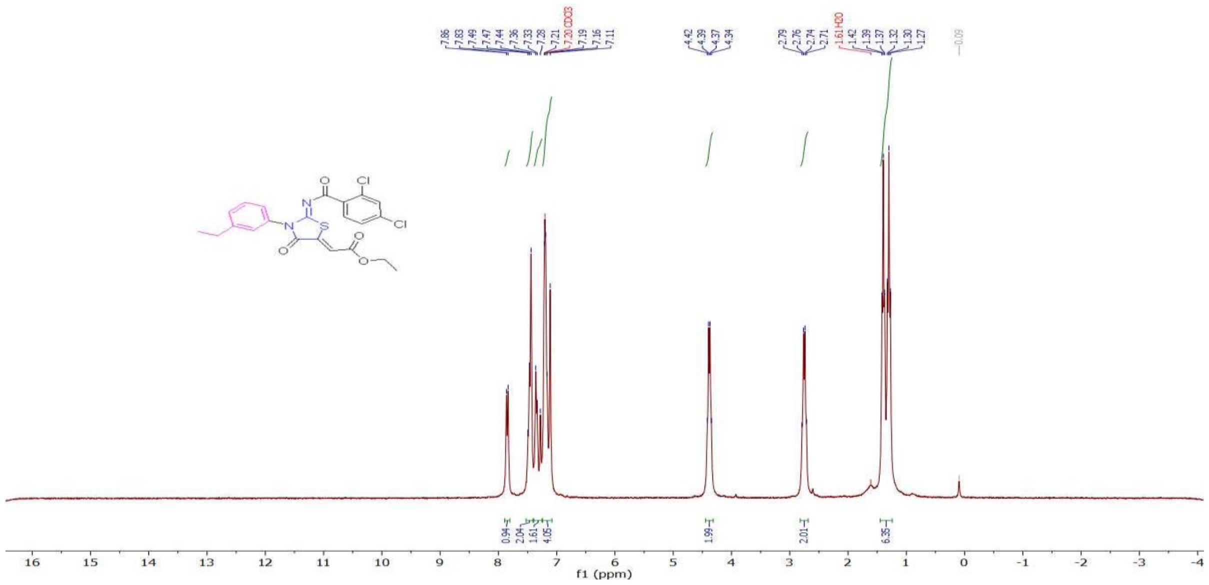

Figure S5 ¹HNMR of Ethyl (Z)-2-((Z)-2-((2,4-dichlorobenzoyl)imino)-3-(3-ethylphenyl)-4-oxothiazolidin-5-ylidene)acetate (6b)

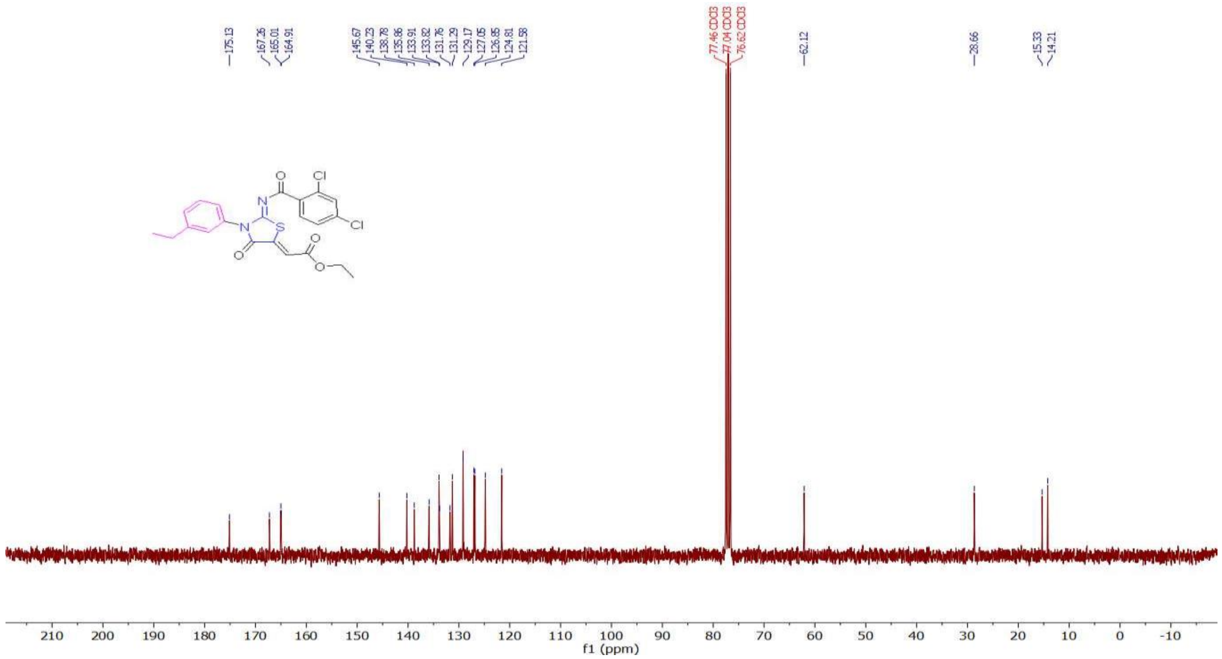

Figure S6 ¹³CNMR of Ethyl (Z)-2-((Z)-2-((2,4-dichlorobenzoyl)imino)-3-(3-ethylphenyl)-4-oxothiazolidin-5-ylidene)acetate (6b)

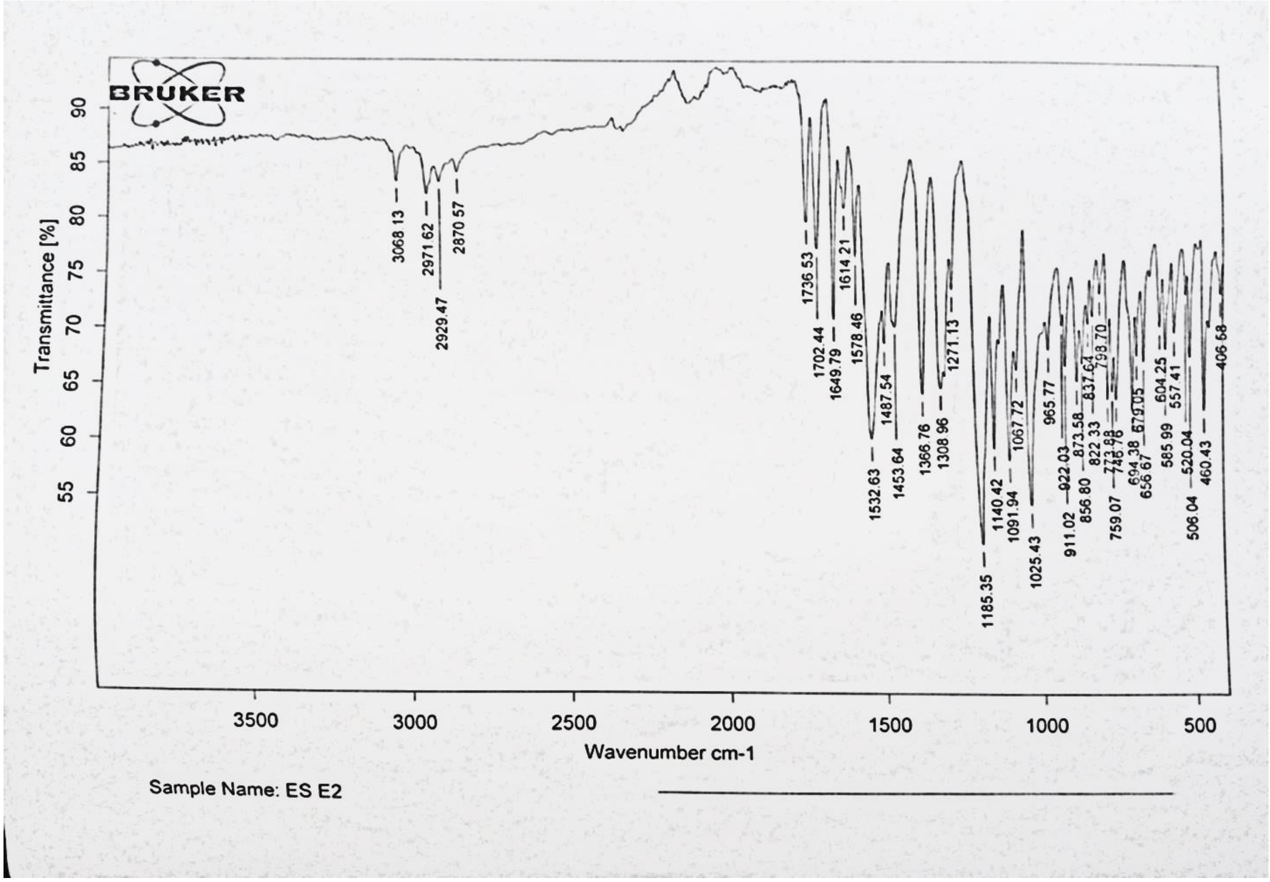

Figure S7 IR spectra of Ethyl (Z)-2-((Z)-2-((2,4-dichlorobenzoyl)imino)-3-(3-ethylphenyl)-4-oxothiazolidin-5-ylidene)acetate (6b)

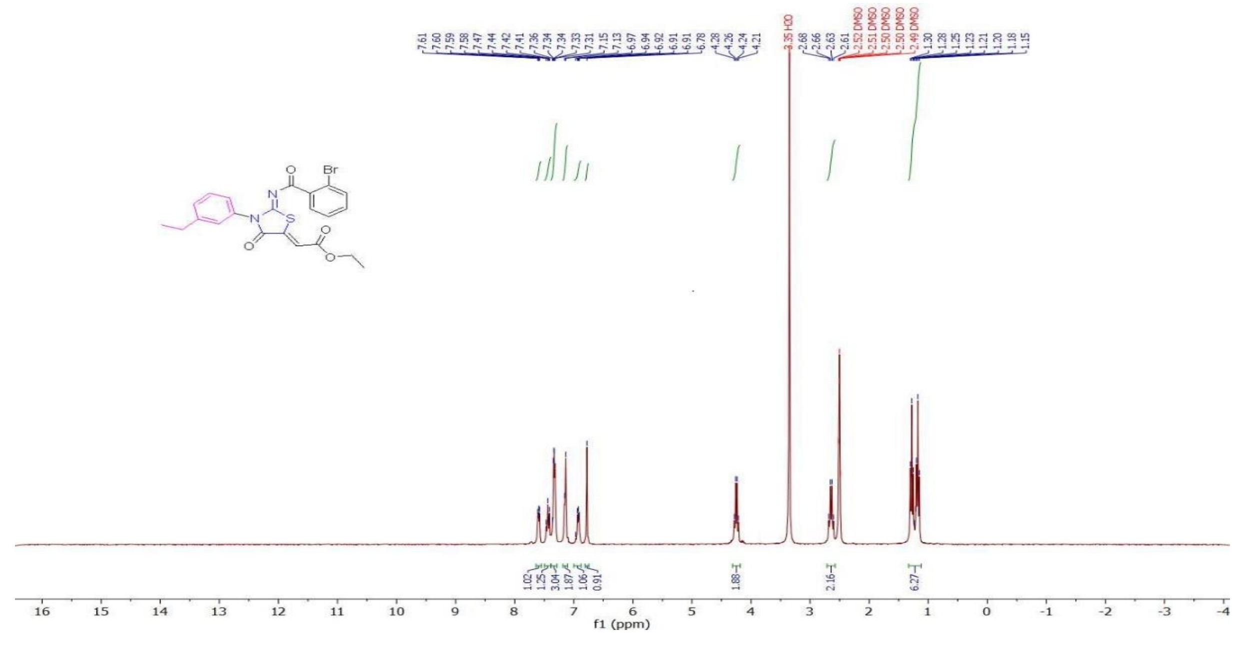

Figure S8 ^{1}HNMRof Ethyl (Z)-2-((Z)-2-((3-bromobenzoyl)imino)-3-(3-ethylphenyl)-4-oxothiazolidin-5-ylidene)acetate (6c)

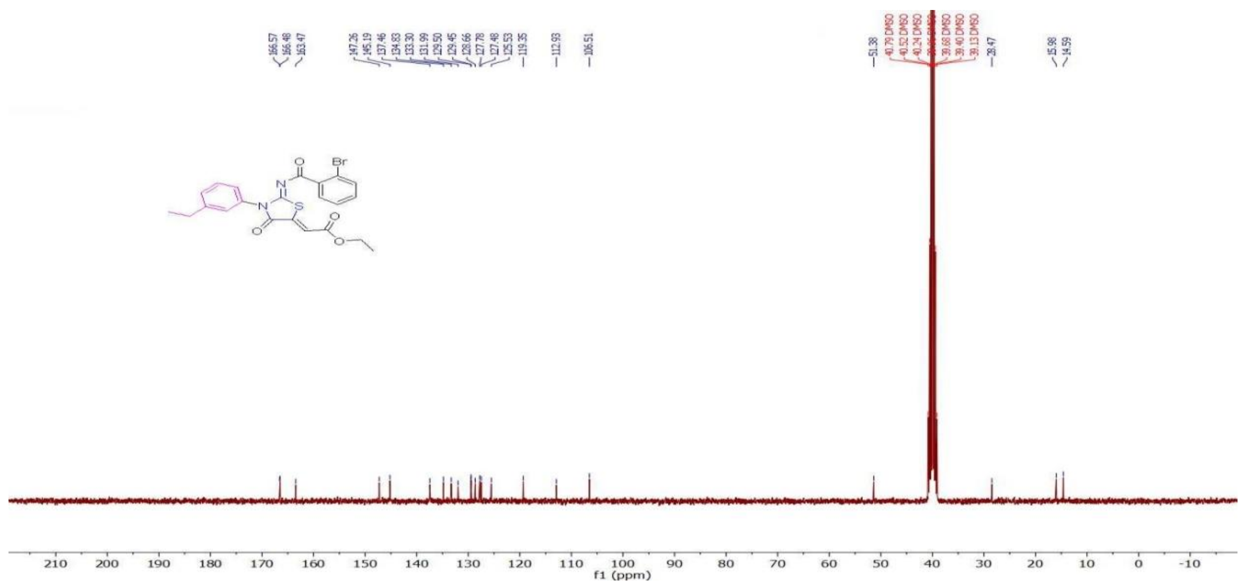

Figure S9 ^{13}CNMR Ethyl (Z)-2-((Z)-2-((3-bromobenzoyl)imino)-3-(3-ethylphenyl)-4-oxothiazolidin-5-ylidene)acetate (6c)

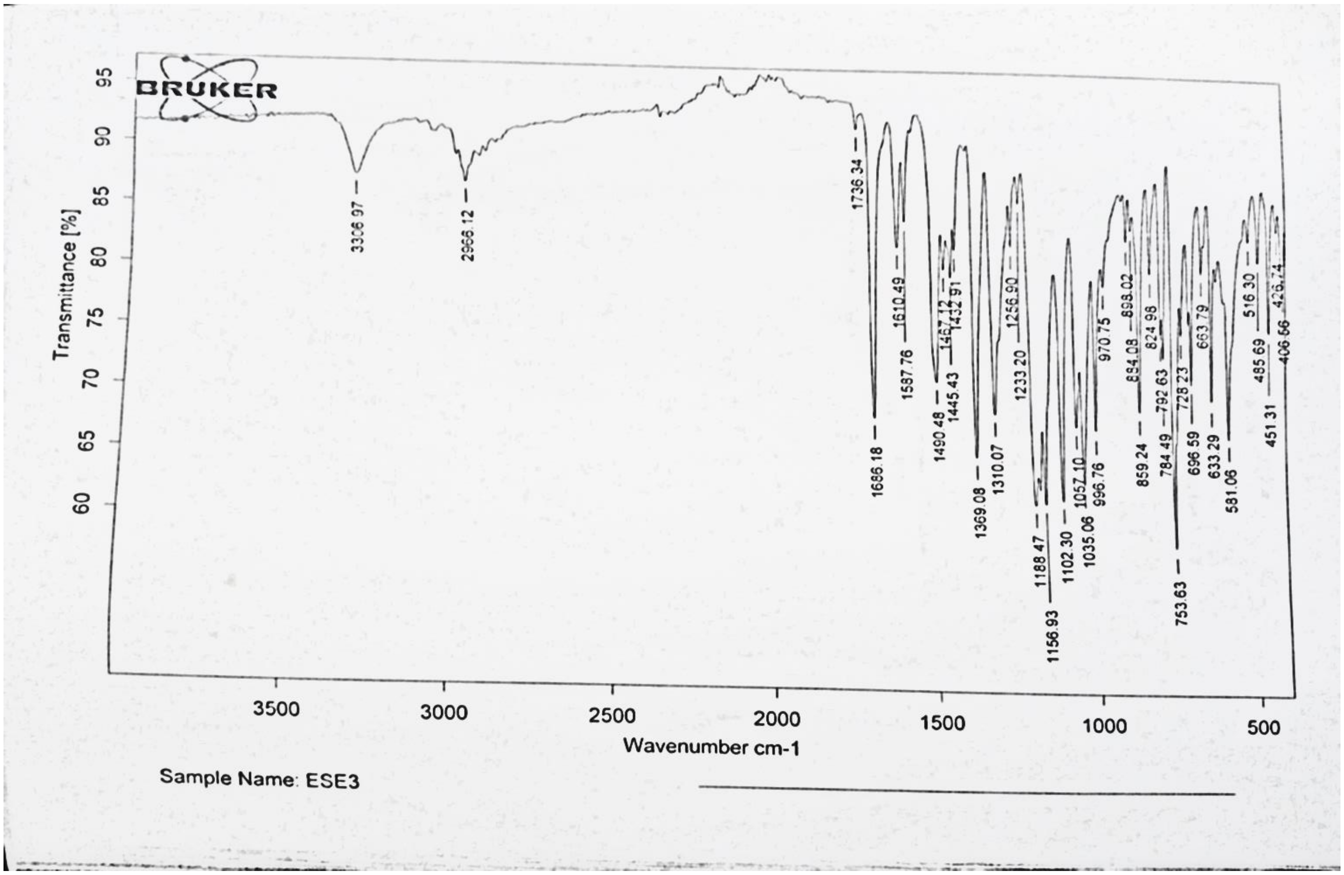

Figure S10 IR spectra of Ethyl (Z)-2-((Z)-2-((3-bromobenzoyl)imino)-3-(3-ethylphenyl)-4-oxothiazolidin-5-ylidene)acetate (6c)

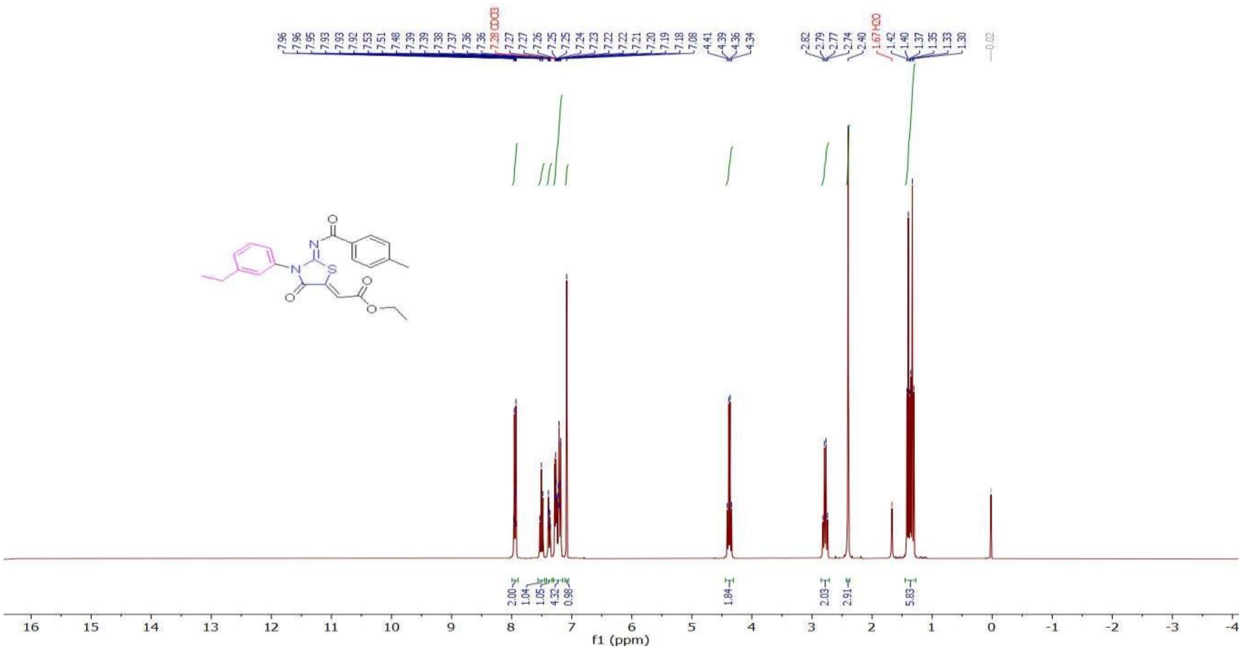

Figure S11 ***1HNMRof Ethyl (Z)-2-((Z)-3-(3-ethylphenyl)-2-((4-methylbenzoyl)imino)-4-oxothiazolidin-5-ylidene)acetate(6d)***

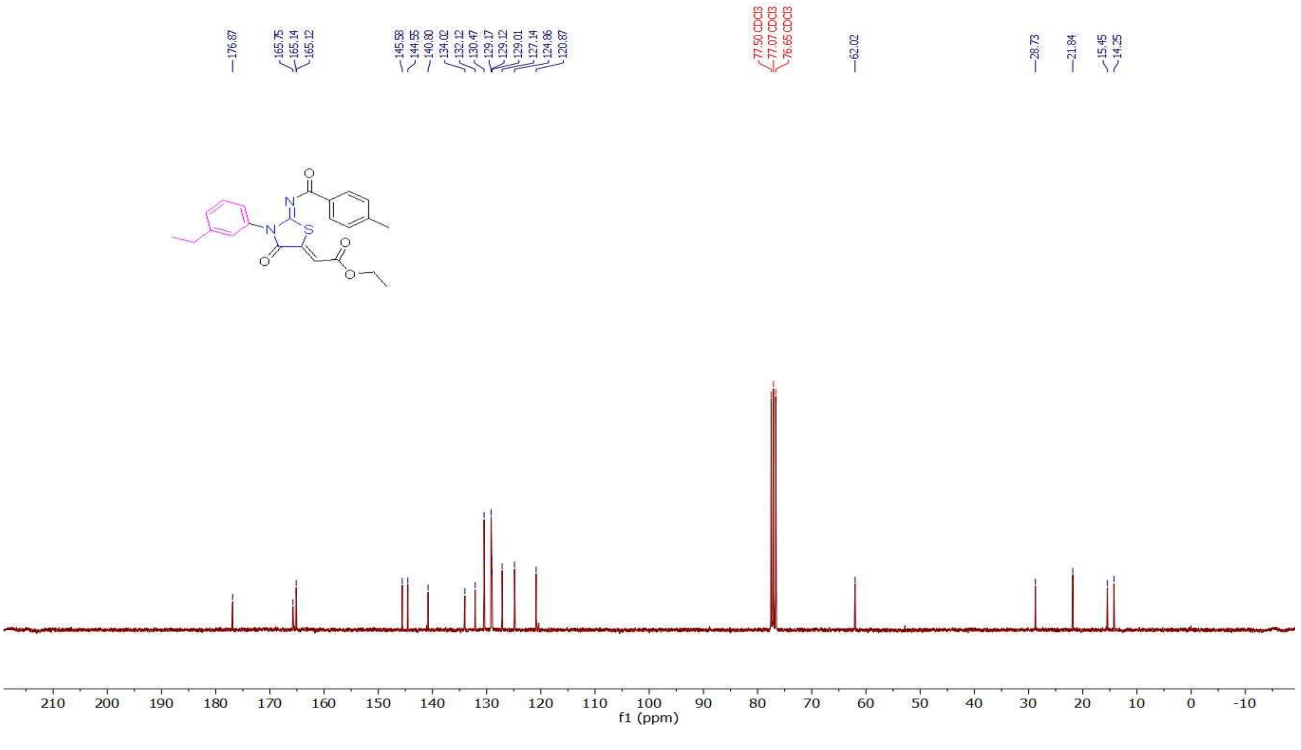

Figure S12 ***13CNMR of Ethyl (Z)-2-((Z)-3-(3-ethylphenyl)-2-((4-methylbenzoyl)imino)-4-oxothiazolidin-5-ylidene)acetate(6d)***

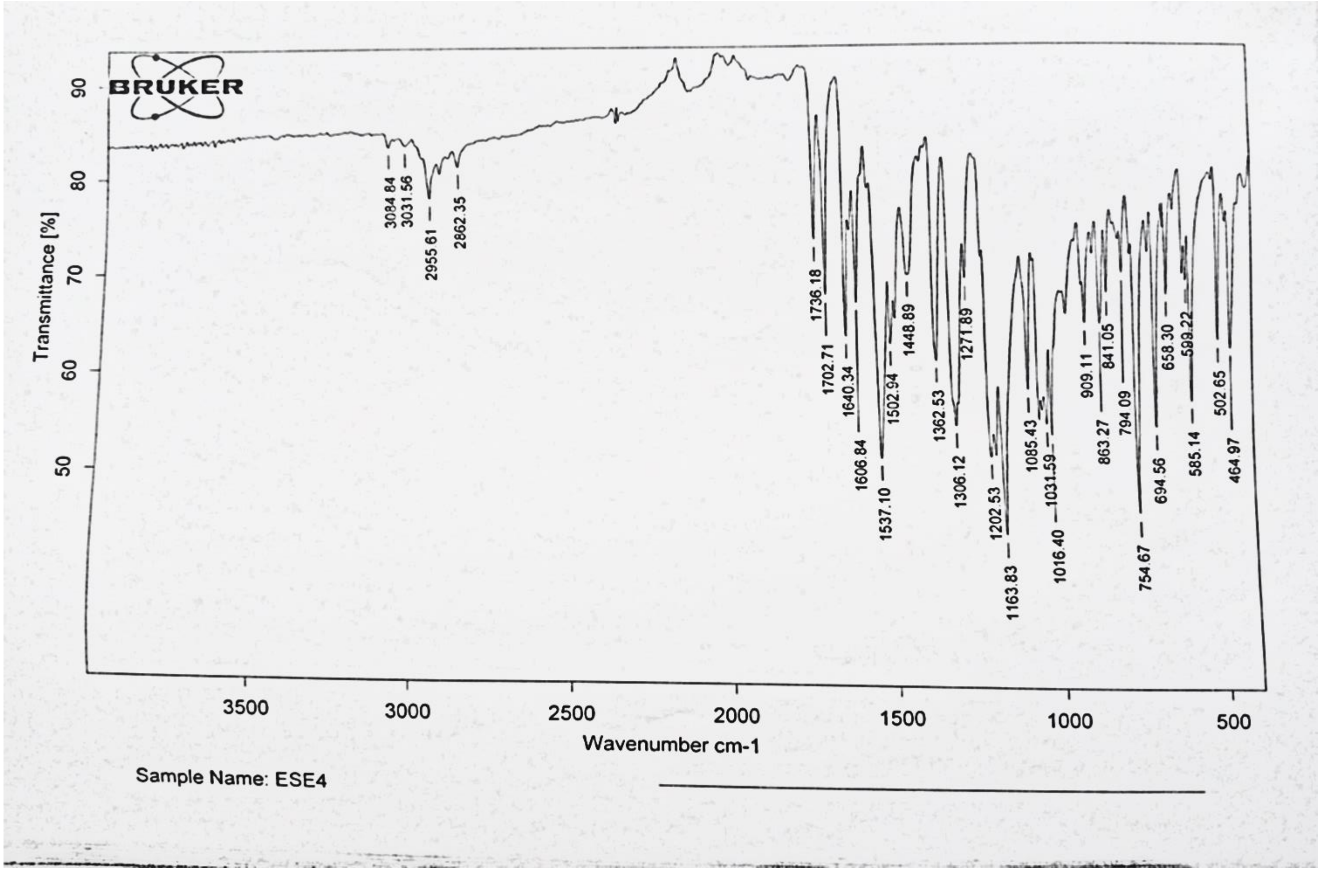

Figure S13 IR spectra of Ethyl (Z)-2-((Z)-3-(3-ethylphenyl)-2-((4-methylbenzoyl)imino)-4-oxothiazolidin-5-ylidene)acetate(6d)

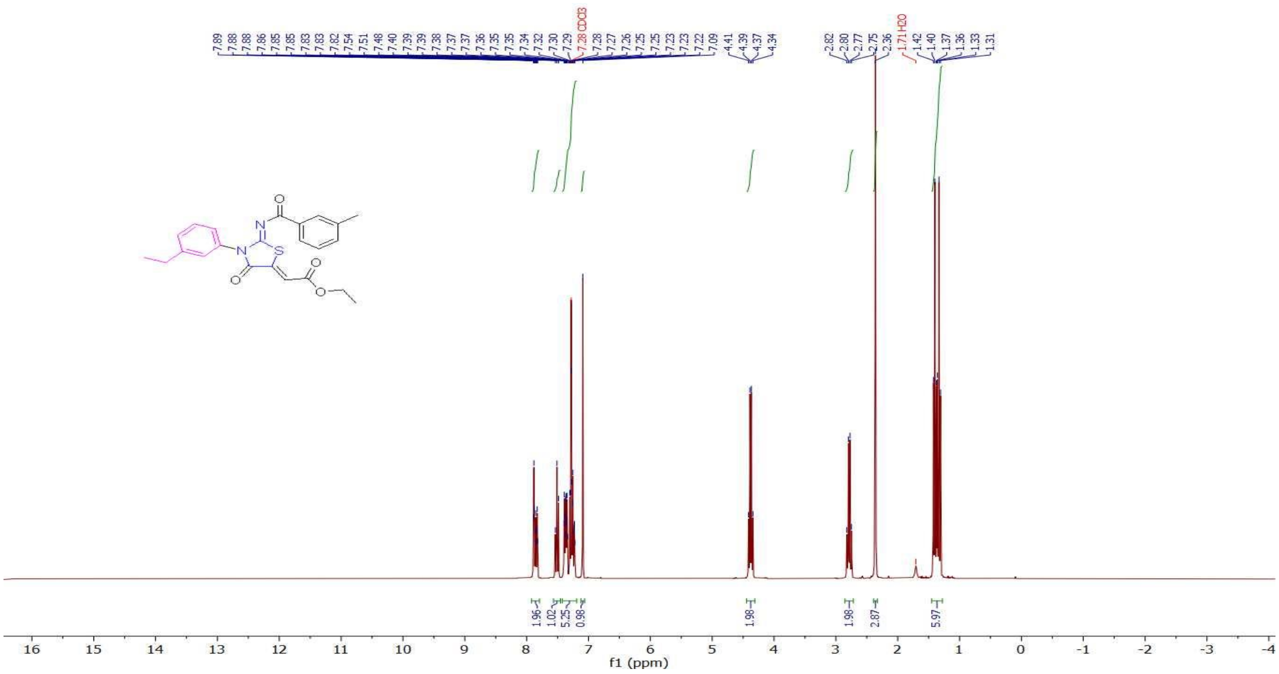

Figure S14 ^{1}HNMR Ethyl (Z)-2-((Z)-3-(3-ethylphenyl)-2-((3-methylbenzoyl)imino)-4-oxothiazolidin-5-ylidene)acetate (6e)

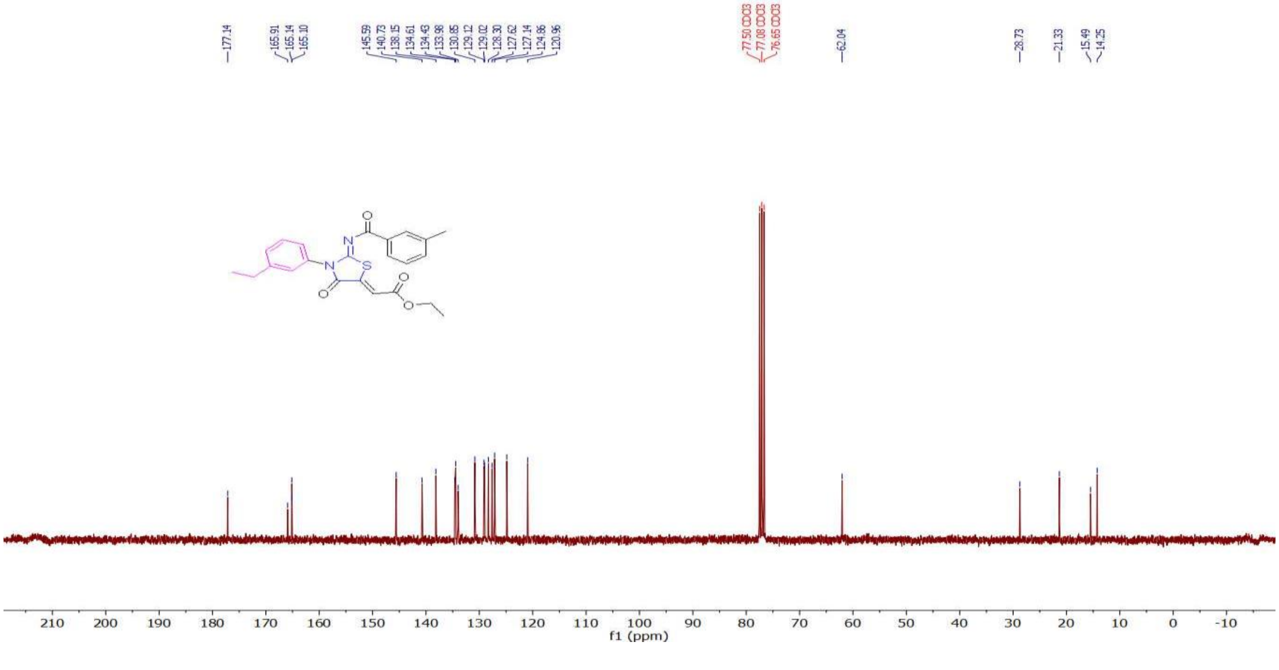

Figure S15 ^{13}CNMR of Ethyl (Z)-2-((Z)-3-(3-ethylphenyl)-2-((3-methylbenzoyl)imino)-4-oxothiazolidin-5-ylidene)acetate (6e)

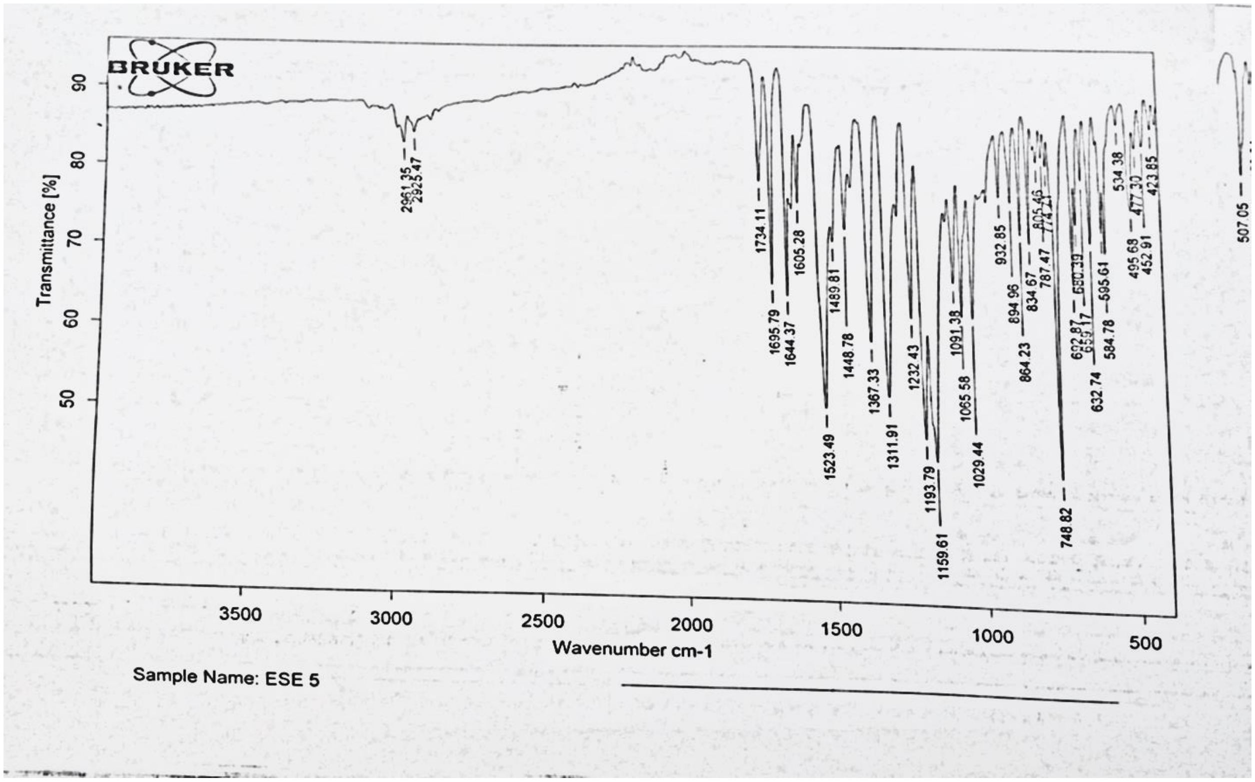

Figure S16 IR spectra of Ethyl (Z)-2-((Z)-3-(3-ethylphenyl)-2-((3-methylbenzoyl)imino)-4-oxothiazolidin-5-ylidene)acetate (6e)

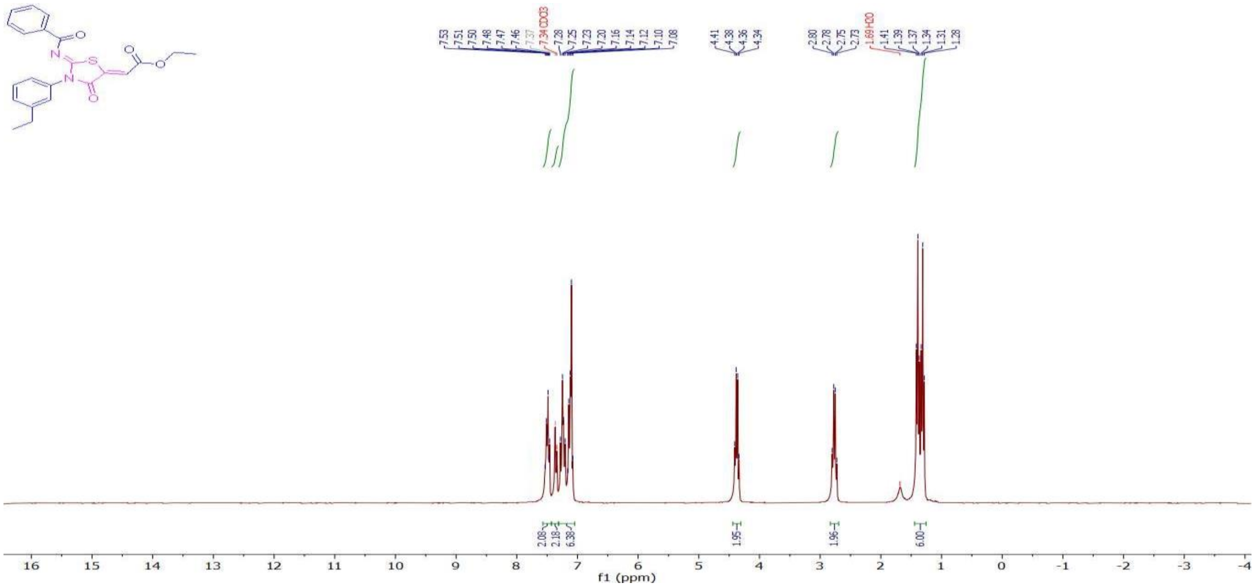

Figure S17 ^{1}HNMR of Ethyl(Z)-2-((Z)-2-(benzoylimino)-3-(3-ethylphenyl)-4-oxothiazolidin-5-ylidene)acetate (6f)

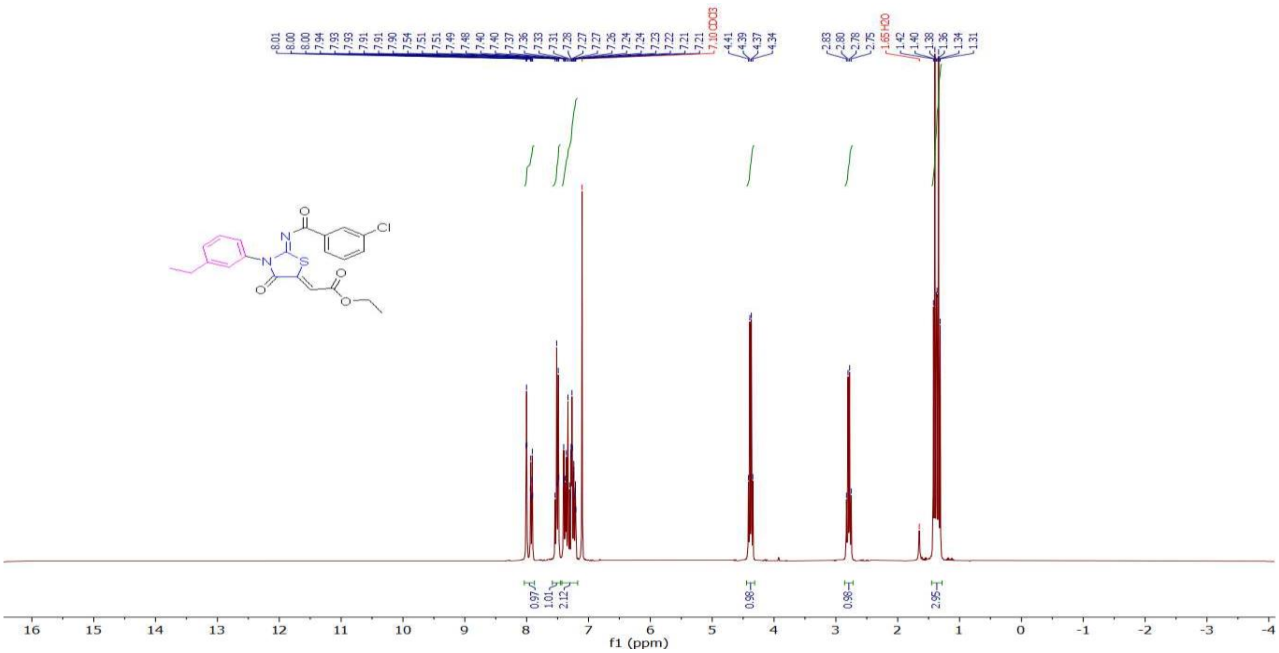

Figure S18 ^{1}HNMR of Ethyl (Z)-2-((Z)-2-((3-chlorobenzoyl)imino)-3-(3-ethylphenyl)-4-oxothiazolidin-5-ylidene)acetate (6g)

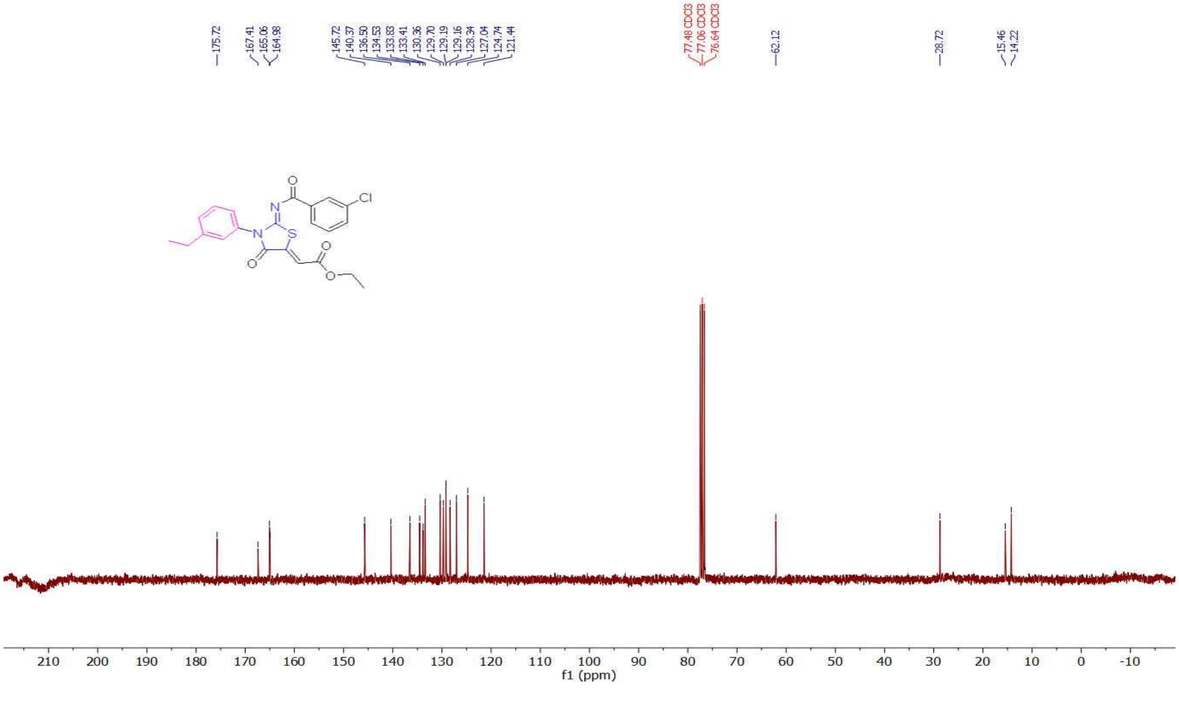

Figure S19 ^{13}CNMR of Ethyl (Z)-2-((Z)-2-((3-chlorobenzoyl)imino)-3-(3-ethylphenyl)-4-oxothiazolidin-5-ylidene)acetate (6g)

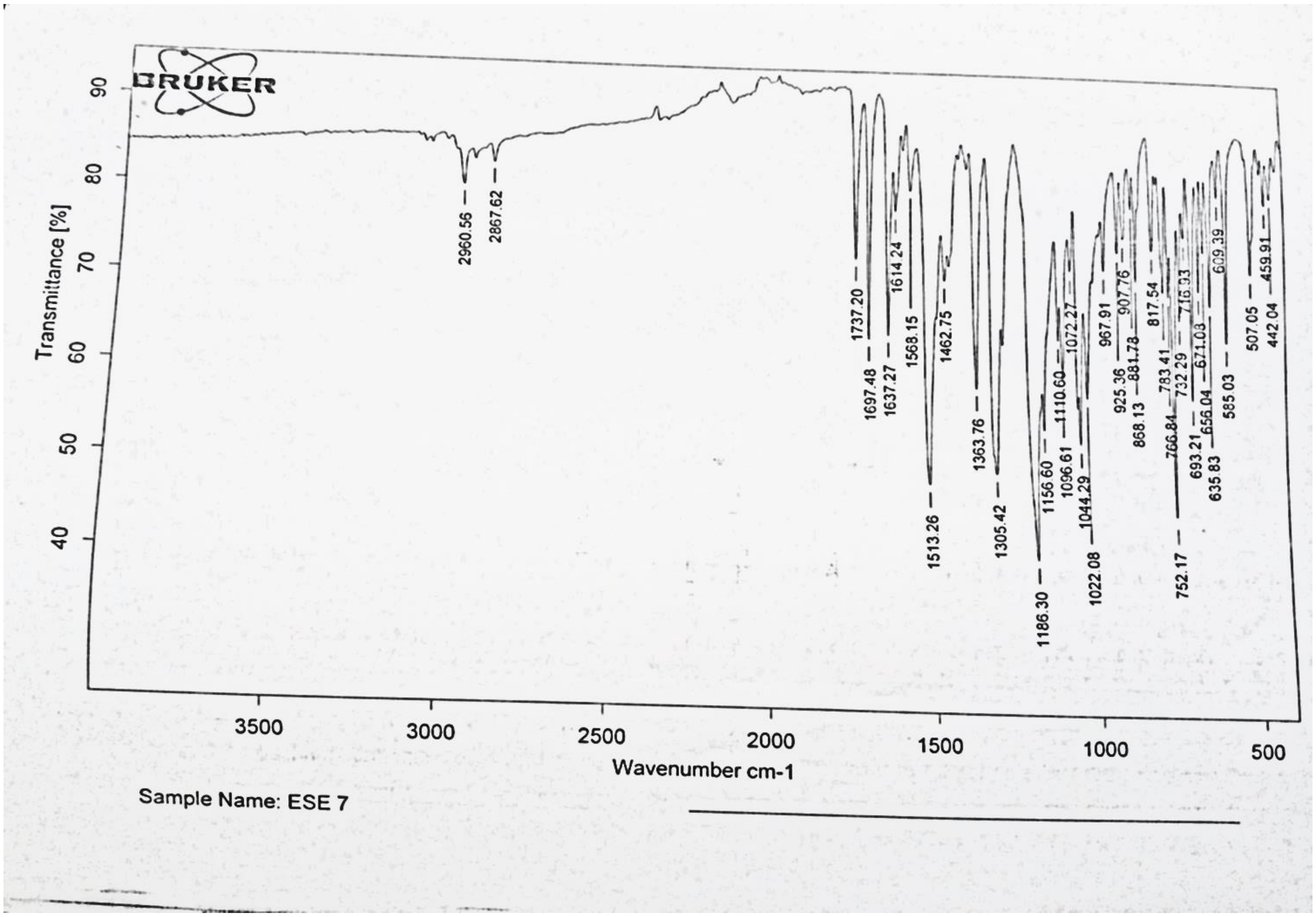

Figure S20 IR spectra of Ethyl (Z)-2-((Z)-2-((3-chlorobenzoyl)imino)-3-(3-ethylphenyl)-4-oxothiazolidin-5-ylidene)acetate (6g)

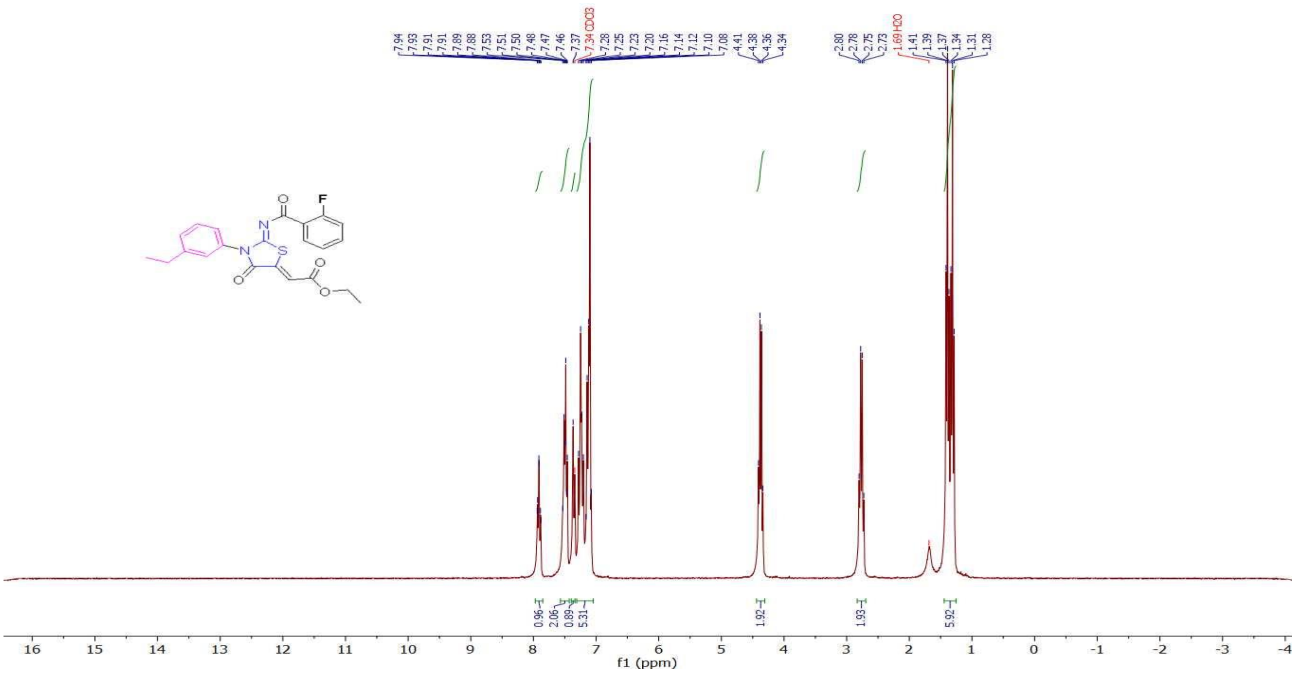

Figure S21 ^{1}HNMR of Ethyl (Z)-2-((Z)-3-(3-ethylphenyl)-2-((2-fluorobenzoyl)imino)-4-oxothiazolidin-5-ylidene)acetate (6h)

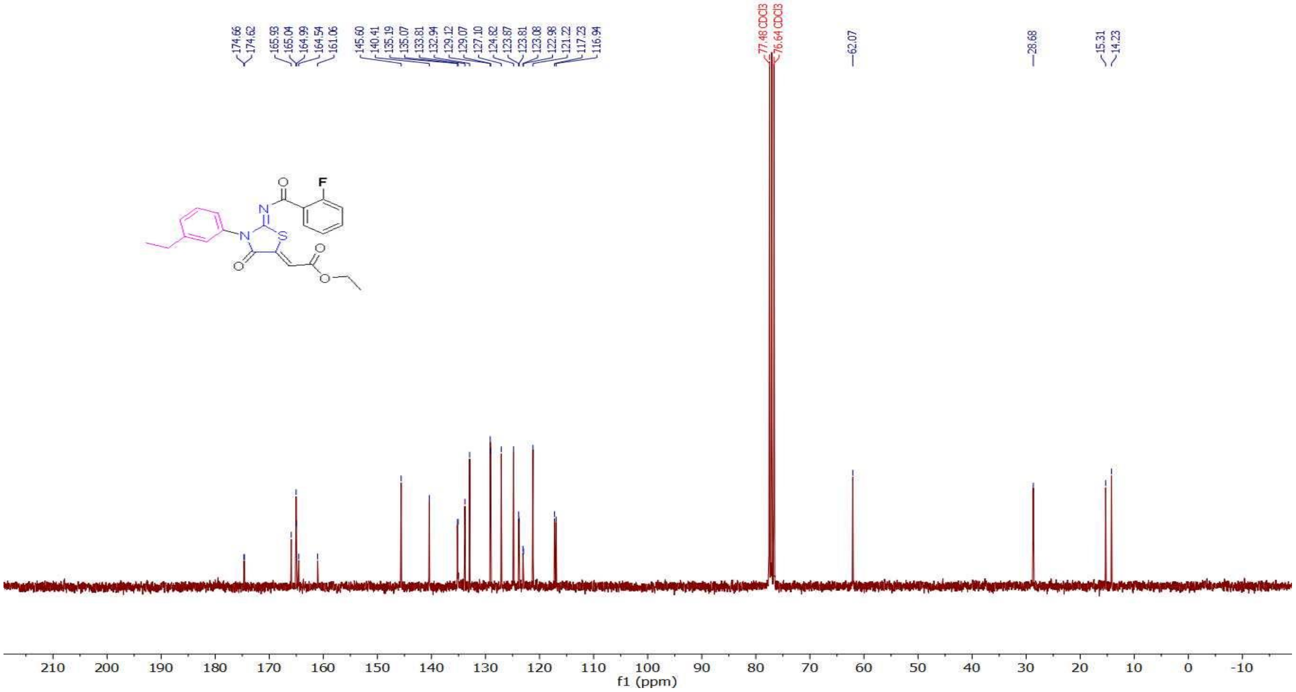

Figure S22 ^{13}CNMR of Ethyl (Z)-2-((Z)-3-(3-ethylphenyl)-2-((2-fluorobenzoyl)imino)-4-oxothiazolidin-5-ylidene)acetate (6h)

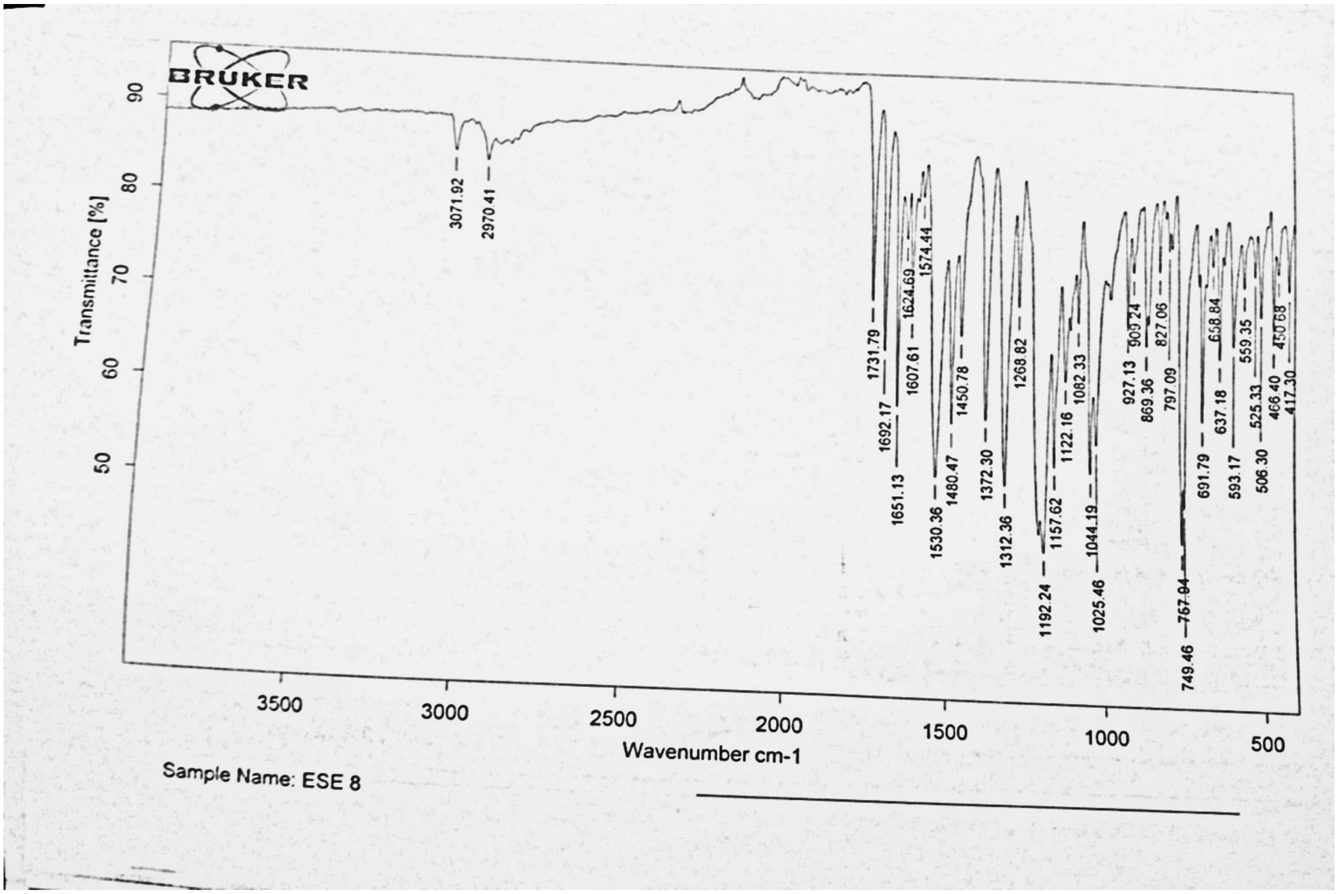

Figure S23 IR spectra of Ethyl (Z)-2-((Z)-3-(3-ethylphenyl)-2-((2-fluorobenzoyl)imino)-4-oxothiazolidin-5-ylidene)acetate (6h)

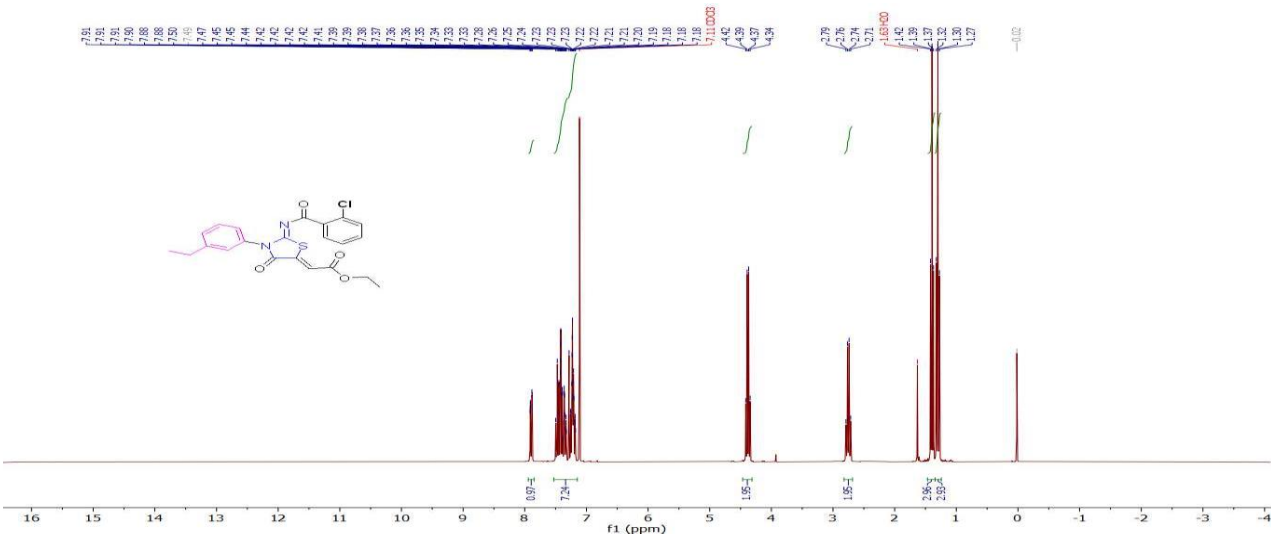

Figure S24 ^{1}HNMR of Ethyl (Z)-2-((Z)-2-((2-chlorobenzoyl)imino)-3-(3-ethylphenyl)-4-oxothiazolidin-5-ylidene)acetate (6i)

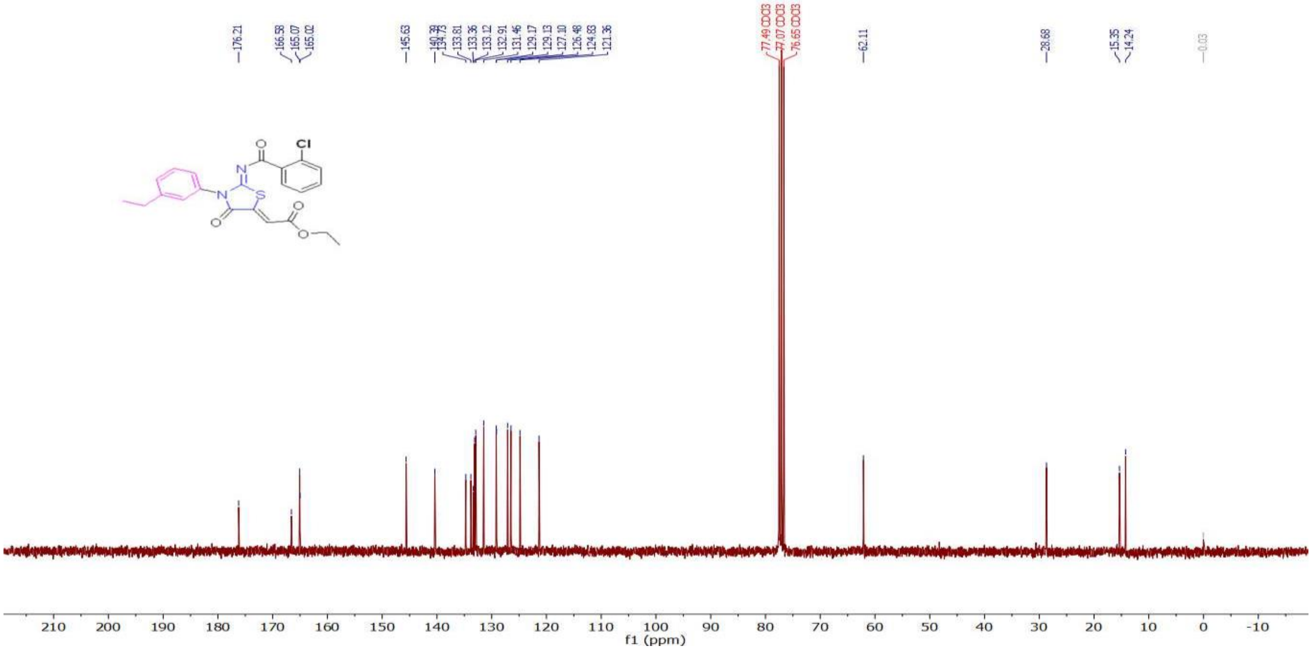

Figure S25 ^{13}CNMR of Ethyl (Z)-2-((Z)-2-((2-chlorobenzoyl)imino)-3-(3-ethylphenyl)-4-oxothiazolidin-5-ylidene)acetate (6i)

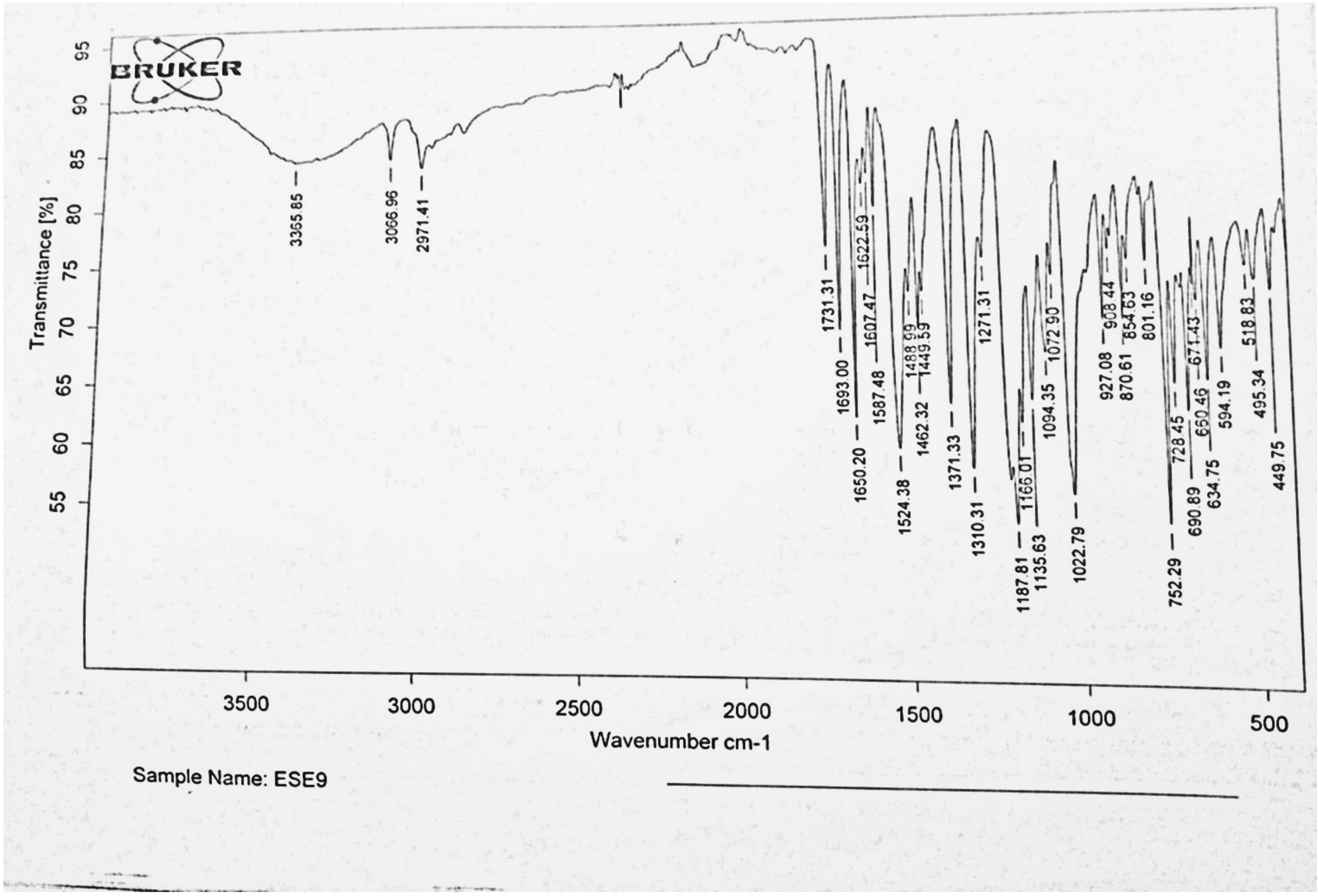

Figure S26 IR spectra of Ethyl (Z)-2-((Z)-2-((2-chlorobenzoyl)imino)-3-(3-ethylphenyl)-4-oxothiazolidin-5-ylidene)acetate (6i)

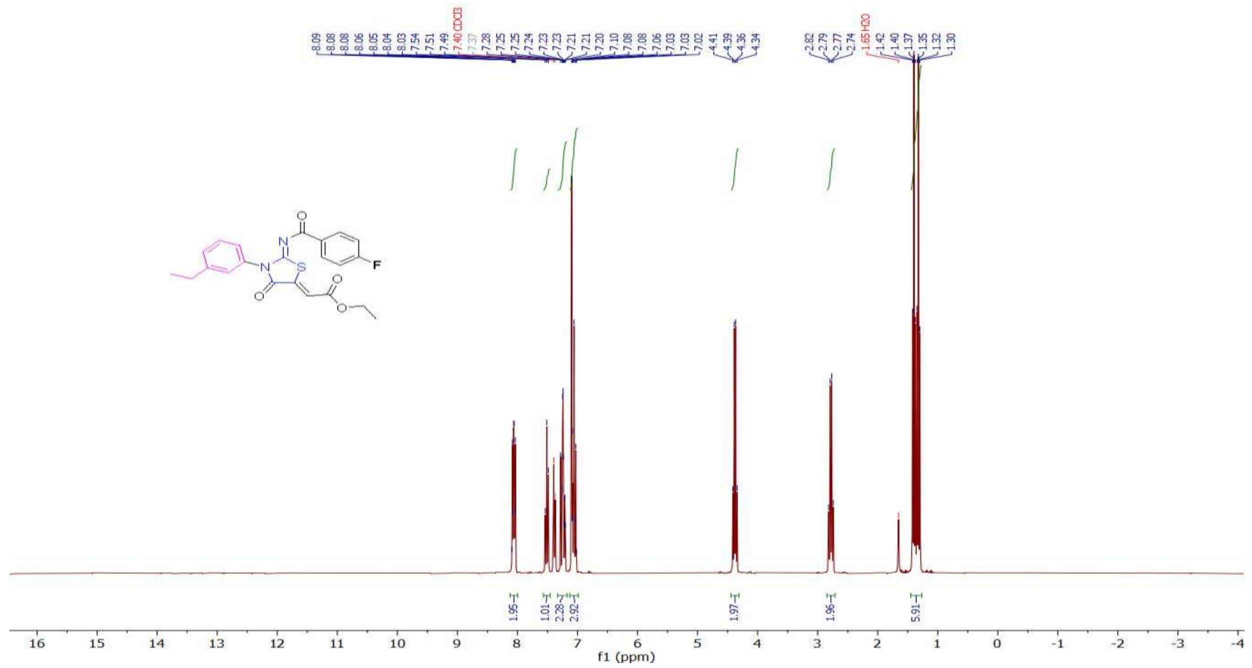

Figure S27 ***^{1}HNMR of Ethyl (Z)-2-((Z)-3-(3-ethylphenyl)-2-((4-fluorobenzoyl)imino)-4-oxothiazolidin-5-ylidene)acetate (6j)***

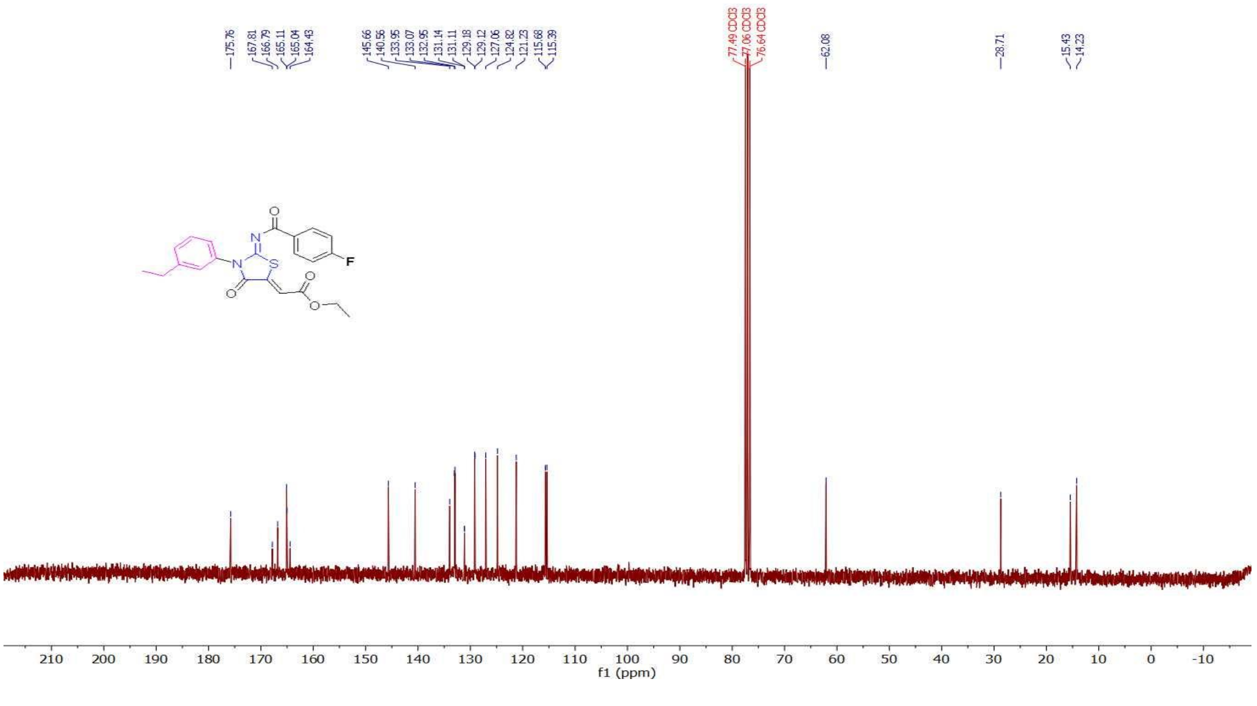

Figure S28 ***^{13}CNMR of Ethyl (Z)-2-((Z)-3-(3-ethylphenyl)-2-((4-fluorobenzoyl)imino)-4-oxothiazolidin-5-ylidene)acetate (6j)***

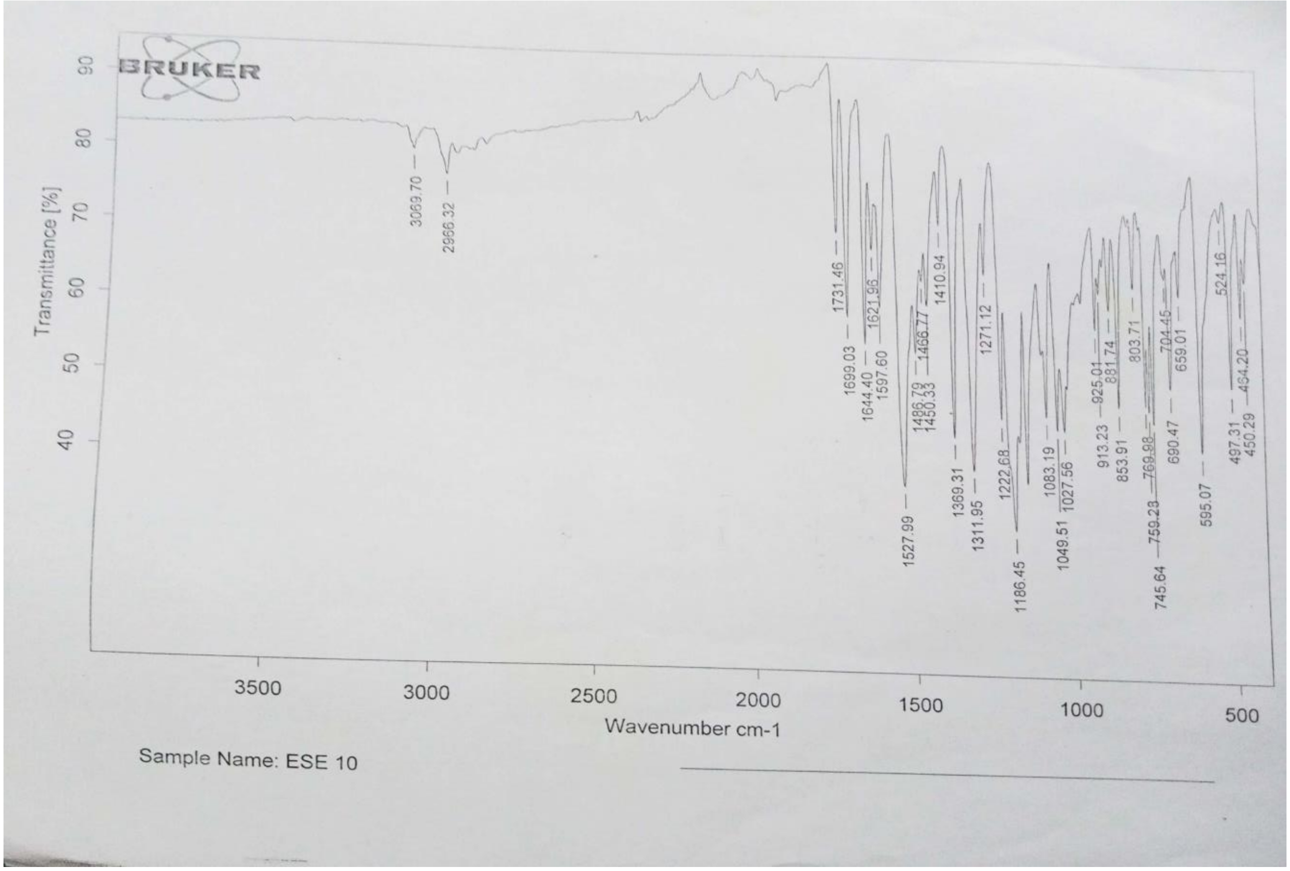

Figure S29 IR spectra of Ethyl (Z)-2-((Z)-3-(3-ethylphenyl)-2-((4-fluorobenzoyl)imino)-4-oxothiazolidin-5-ylidene)acetate (6j)

Optimized structures

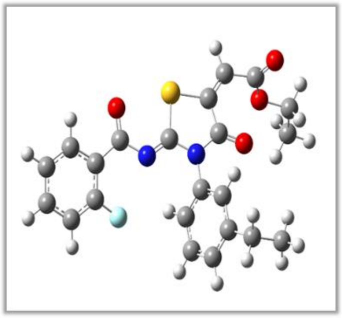

6b

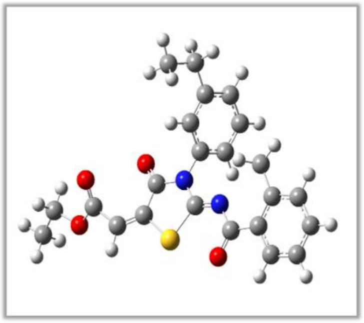

6c

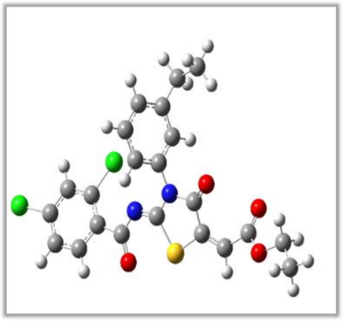

6d

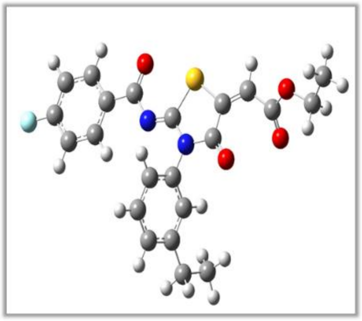

6f

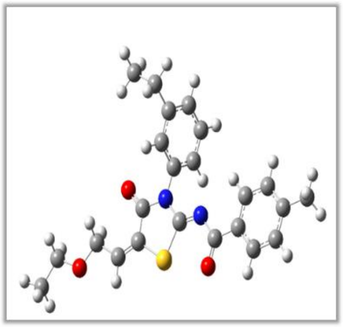

6h

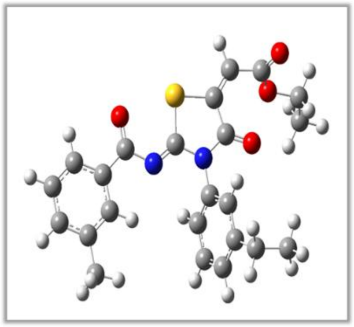

6i

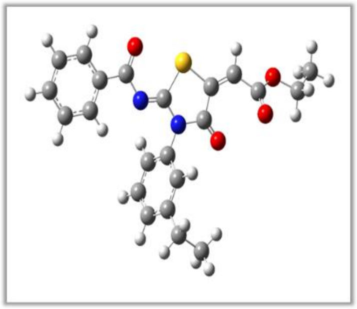

6j

Figure S30. Showing the optimized structure of remaining compounds

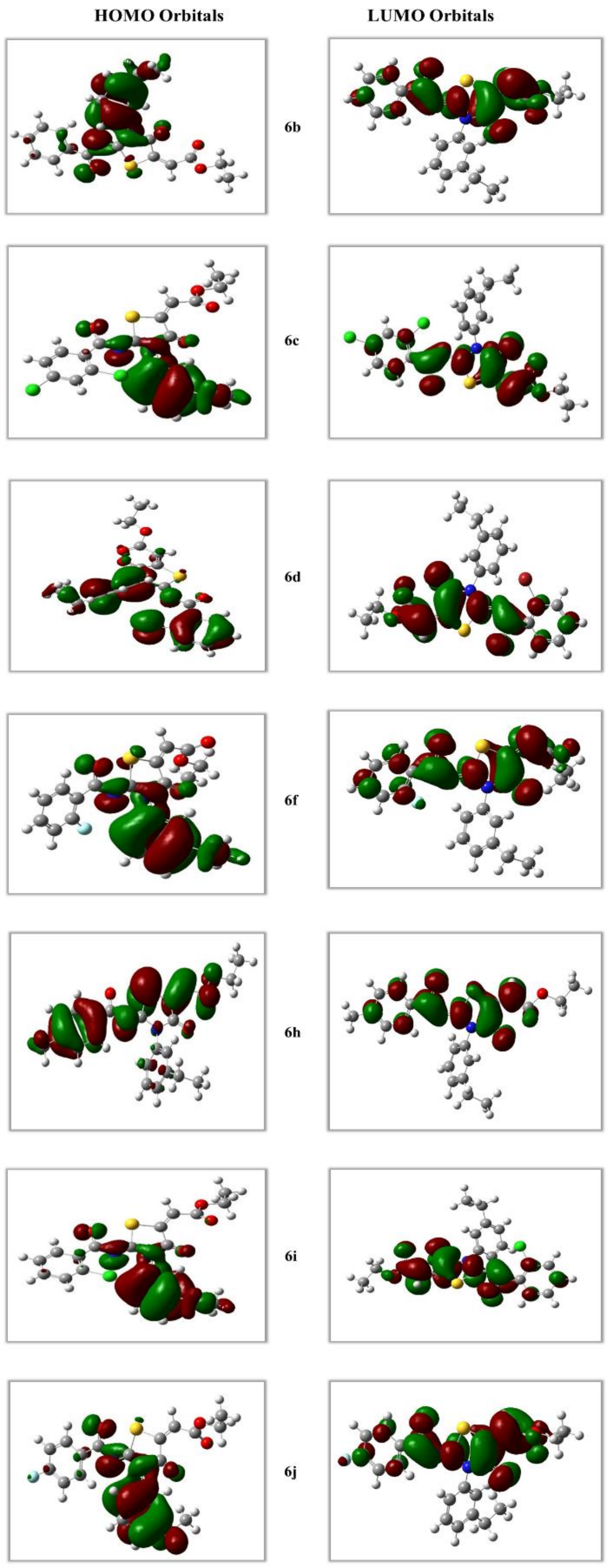

Figure S31. Representing the HOMO-LUMO Visualization

Molecular Docking studies

The compound 6f and 6h also demonstrate potential interactions including hydrogen bonding. However, the number of alkyl interactions found in **6f** are less than **6g** and **6e** making it less potent. It can be concluded that this Pi-Cation interaction is responsible for higher activities of **6e** and **6g.** Compounds **6h, 6i** and **6j** showed comparable bonding pattern up to some extent reflecting their equipotency towards carbonic anhydrase inhibition potency. In compounds, **6c, 6j** and **6i** carbonyl oxygen formed two hydrogen bonds with amino acid residue THR 199 and THR 200.while in compounds **6b, 6h** and **6j** alkyl interactions involving amino acid residues HIS94; HIS119; VAL121; VAL143; and TRP209 were observed. However in **6i**, a metal acceptor interaction between oxygen atom of ethoxy group present at the terminal position and Zn atom was also formed contributing to stability of ligand protein binding.In compound **6d**, Pi-Alkyl interactions of aromatic and thiazolidine rings was contributing the stability of ligand protein complex.

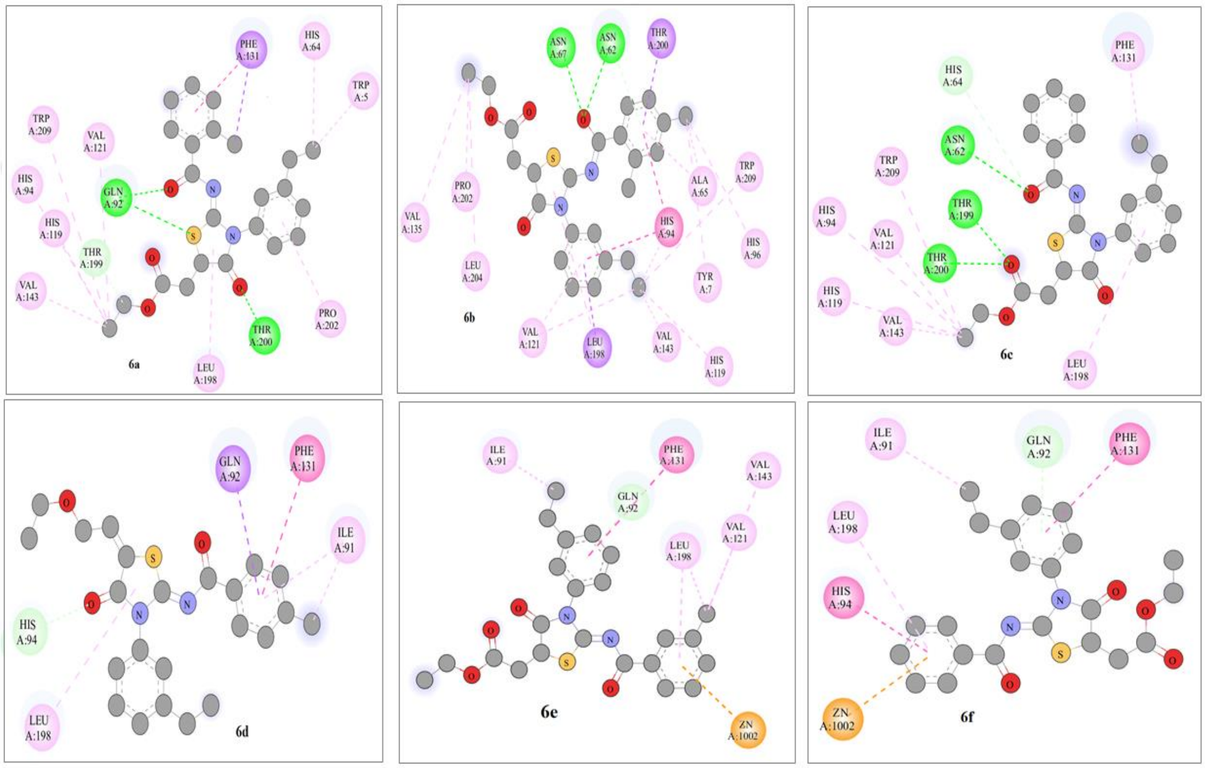

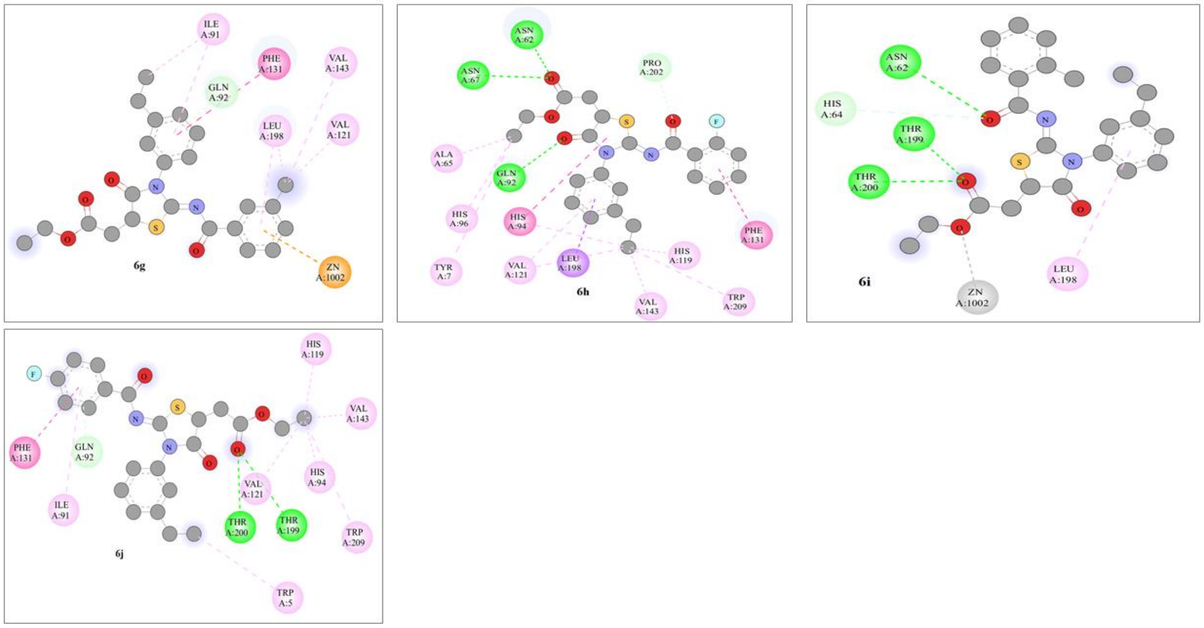

Figure S32. Molecular interactions of synthesized derivatives

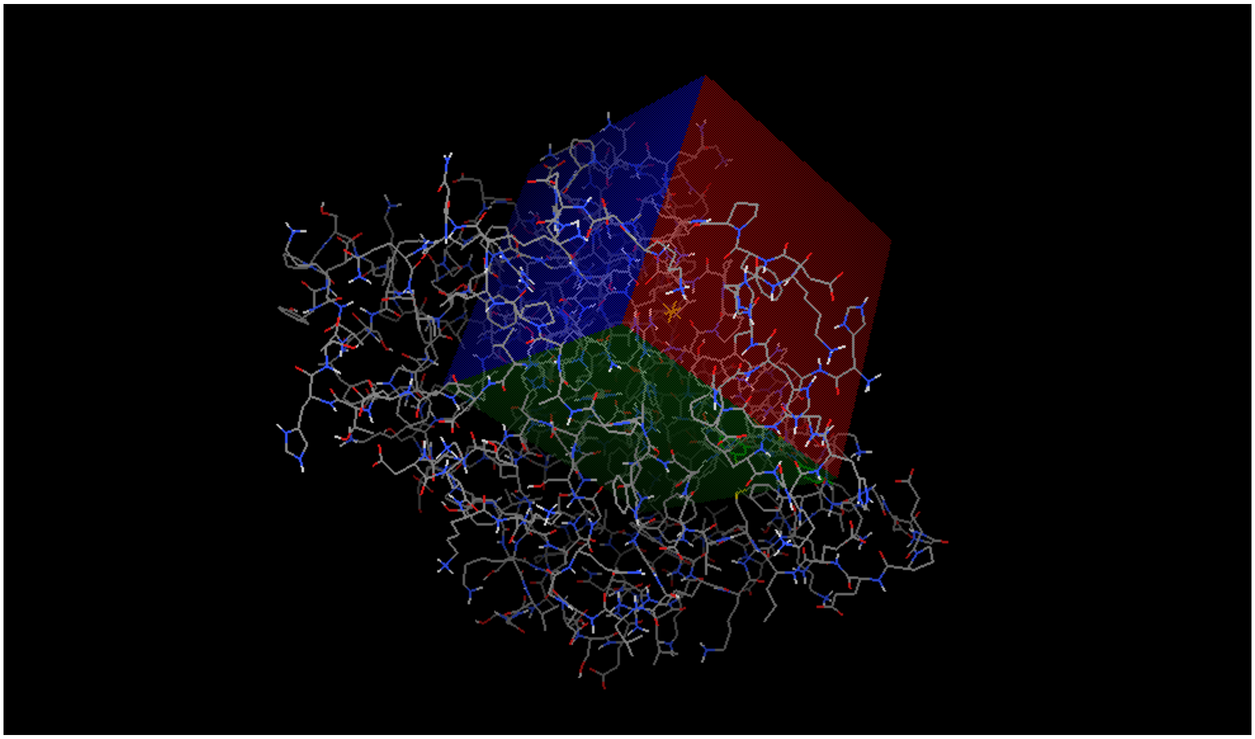

Figure S33. Grid box

Table S1 ***Corelation of the computational results and experimental data***

Correlation coefficent was identified using statistical package of social sciences and found statistically significant as there is significant positive relationship between in-vitro activity and computational activities of compound. For example, compound **6a, 6e and 6g** had strong in-vitro activites and ultimately there is high docking scores obtained from computational work.

SUMMARY OUTPUT

Regression Statistics	
Multiple R	0.982576
R Square	0.965455
Adjusted R Square	0.95394
Standard Error	0.804297
Observations	5

ANOVA

	df	*SS*	*MS*	*F*	*Significance F*
Regression	1	54.23804	54.23804	83.84375638	0.002754
Residual	3	1.940683	0.646894		
Total	4	56.17872			

	Coefficients	*Standard Error*	*t Stat*	*P-value*	*Lower 95%*	*Upper 95%*	*Lower 95.0%*	*Upper 95.0%*
Intercept	-6.52814	0.423915	-15.3996	0.000594822	-7.87722	-5.17905	-7.87722	-5.17905
in-vitro activity	0.143654	0.015689	9.156624	0.002753752	0.093726	0.193582	0.093726	0.193582
